# Interlayer Engineering of α‐MoO_3_ Modulates Selective Hydronium Intercalation in Neutral Aqueous Electrolyte

**DOI:** 10.1002/anie.202010073

**Published:** 2020-11-09

**Authors:** Haozhe Zhang, Weixing Wu, Qiyu Liu, Fan Yang, Xin Shi, Xiaoqing Liu, Minghao Yu, Xihong Lu

**Affiliations:** ^1^ MOE of the Key Laboratory of Bioinorganic and Synthetic Chemistry The Key Lab of Low-carbon Chem & Energy Conservation of Guangdong Province School of Chemistry Sun Yat-sen University Guangzhou 510275 P. R. China; ^2^ Center for Advancing Electronics Dresden (cfaed) & Faculty of Chemistry and Food Chemistry Technische Universität Dresden Dresden 01062 Germany

**Keywords:** aqueous batteries, hydronium intercalation, interlayer engineering, zinc metal batteries, α-MoO_3_

## Abstract

Among various charge‐carrier ions for aqueous batteries, non‐metal hydronium (H_3_O^+^) with small ionic size and fast diffusion kinetics empowers H_3_O^+^‐intercalation electrodes with high rate performance and fast‐charging capability. However, pure H_3_O^+^ charge carriers for inorganic electrode materials have only been observed in corrosive acidic electrolytes, rather than in mild neutral electrolytes. Herein, we report how selective H_3_O^+^ intercalation in a neutral ZnCl_2_ electrolyte can be achieved for water‐proton co‐intercalated α‐MoO_3_ (denoted WP‐MoO_3_). H_2_O molecules located between MoO_3_ interlayers block Zn^2+^ intercalation pathways while allowing smooth H_3_O^+^ intercalation/diffusion through a Grotthuss proton‐conduction mechanism. Compared to α‐MoO_3_ with a Zn^2+^‐intercalation mechanism, WP‐MoO_3_ delivers the substantially enhanced specific capacity (356.8 vs. 184.0 mA h g^−1^), rate capability (77.5 % vs. 42.2 % from 0.4 to 4.8 A g^−1^), and cycling stability (83 % vs. 13 % over 1000 cycles). This work demonstrates the possibility of modulating electrochemical intercalating ions by interlayer engineering, to construct high‐rate and long‐life electrodes for aqueous batteries.

## Graphical abstract

Selective H_3_O^+^ intercalation is demonstrated for a water‐proton co‐intercalated α‐MoO_3_ cathode in a neutral ZnCl_2_ electrolyte, thus providing substantially enhanced specific capacity, rate capability, and cycling stability. H_2_O molecules between the α‐MoO_3_ interlayers are uncovered to block Zn^2+^ intercalation pathways, while allowing smooth H_3_O^+^ intercalation/diffusion through a Grotthuss proton conduction mechanism.

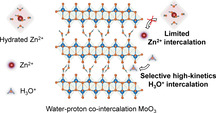

## Introduction

Rechargeable aqueous batteries with neutral electrolytes have attracted intensive scientific attention as promising alternatives for large‐scale energy storage technologies. The utilized water‐based electrolytes offer significant advantages of high ionic conductivity (≈1 S cm^−1^), simplified manufacture, low cost, and intrinsic safety.^[1]^ In particular, Zn metal batteries (ZMBs) with mild aqueous electrolytes have recently stood out, due to the direct use of Zn metal anodes with a high specific capacity (≈820 mA h g^−1^) and a low stripping/plating potential (−0.76 V vs. standard hydrogen electrode).^[2]^ Numerous efforts have been devoted to exploring Zn^2+^‐host cathode materials of ZMBs, which has brought Mn‐compounds, V‐compounds, Prussian blue materials into the spotlight.^[3]^ However, the Zn^2+^‐intercalation chemistry generally shows sluggish kinetics and unsatisfactory cycling stability.^[4]^ In aqueous electrolytes, Zn^2+^ tends to form a large‐size hydrated state (Zn(H_2_O)_6_^2+^ with 5.5 Å) due to the strong Zn^2+^‐water interaction.^[5]^ The intercalation of Zn^2+^ into cathode hosts thus requires large de‐solvation and intercalation energy. Besides, bivalent Zn^2+^ imposes a strong repulsive force with the hosts, leading to the large Zn^2+^‐diffusion energy barriers within the hosts and the undesired structure distortion of hosts.^[6]^

Apart from Zn^2+^, non‐metal hydronium (H_3_O^+^) has also been recognized as favorable charge carrier ions for aqueous batteries. Assigned to the small ionic size (≈1.0 Å) and light molecular mass, H_3_O^+^ intercalation presents attractive high‐kinetics and highly reversible behaviors.^[7]^ The partial involvement of H_3_O^+^ intercalation was also discovered for the charge‐storage mechanism of ZMB cathodes.^[8]^ For example, Sun et al. uncovered the consequent intercalation of H_3_O^+^ and Zn^2+^ for ϵ‐MnO_2_ cathode in a mixed ZnSO_4_/MnSO_4_ electrolyte.^[9]^ The charge‐transfer resistance of ϵ‐MnO_2_ in the H_3_O^+^‐intercalation step is three orders of magnitude smaller than that in the Zn^2+^‐intercalation step. A similar phenomenon was also observed for polyaniline‐intercalated MnO_2_ nanolayers, in which the diffusion coefficient of H_3_O^+^ (5.84×10^−12^ cm^2^ s^−1^) was substantially higher than that of Zn^2+^ (7.35×10^−14^ cm^2^ s^−1^).^[10]^ These findings inspire that selective H_3_O^+^ intercalation into cathodes would bring the constructed ZMBs with significant performance advance in terms of capacity, kinetics, as well as cycle life. However, thus far, pure H_3_O^+^‐intercalation behavior for layered/tunneled cathodes has only been observed in corrosive acidic electrolytes.[^7b^, ^11^] It remains a grand challenge to achieve selective H_3_O^+^ intercalation in mild neutral electrolytes.

**Figure 1:**
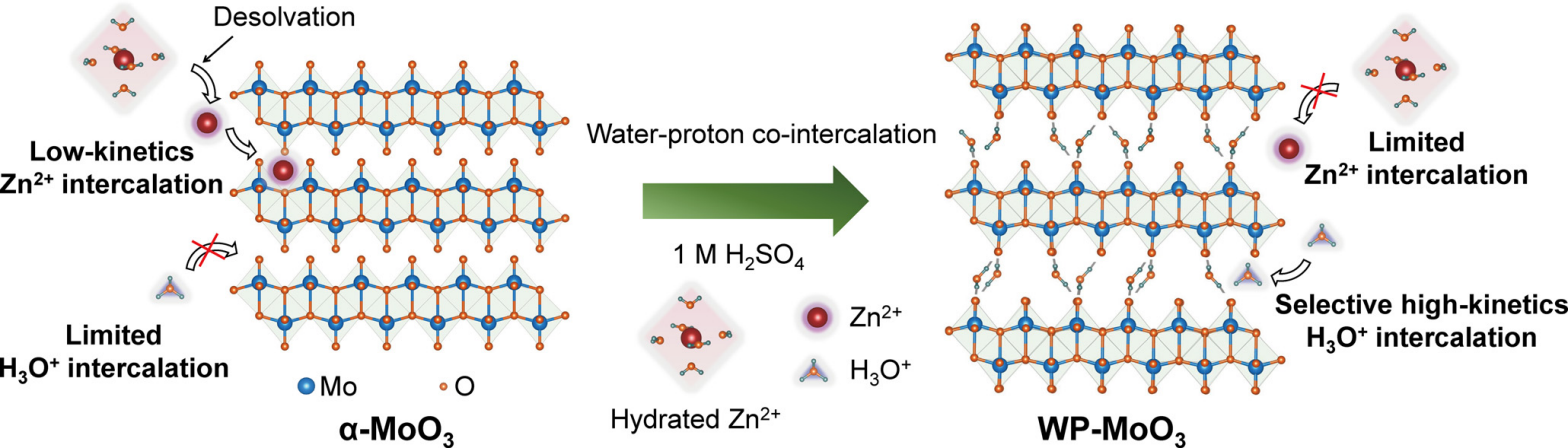
Schematic illustration of the Zn^2+^‐intercalation chemistry for α‐MoO_3_ and the selective H_3_O^+^‐intercalation chemistry for WP‐MoO_3_.

In this study, we, taking orthorhombic MoO_3_ (α‐MoO_3_) as an example, for the first time demonstrate the feasibility of selective H_3_O^+^ intercalation in a neutral ZnCl_2_ electrolyte. α‐MoO_3_ is selected due to its typical layered structure with distorted [MoO_6_] octahedra bilayers weakly bonded by van der Waals force.^[12]^ The complete redox of Mo^4+^/Mo^6+^ allows α‐MoO_3_ with an attractive theoretical capacity of 372 mA h g^−1^. Selective H_3_O^+^‐intercalation chemistry is modulated for α‐MoO_3_ through a water‐proton co‐intercalation strategy (denoted WP‐MoO_3_), which further tackles the low‐capacity, poor‐rate, and short‐life issues faced by pristine α‐MoO_3_ with Zn^2+^‐intercalation chemistry. H_2_O molecules located between WP‐MoO_3_ interlayers impose a high Zn^2+^‐intercalation energy barrier by blocking Zn^2+^ diffusion pathways (Figure 1). Meanwhile, H_3_O^+^ intercalation/diffusion can be smoothly achieved within WP‐MoO_3_ interlayers through a well‐known Grotthuss mechanism (proton jumping between water molecules).^[13]^ In contrast to Zn^2+^‐intercalation α‐MoO_3_, selective H_3_O^+^‐intercalation WP‐MoO_3_ depicts substantially enhanced redox depth (1.92 vs. 0.99 e^−^ per Mo atom; 357 vs. 184 mA h g^−1^), rate capability (77.5 % vs. 42.2 % from 0.4 to 4.8 A g^−1^), and cycling stability (83 % vs. 13 % over 1000 cycles).

## Results and Discussion

**Figure 2:**
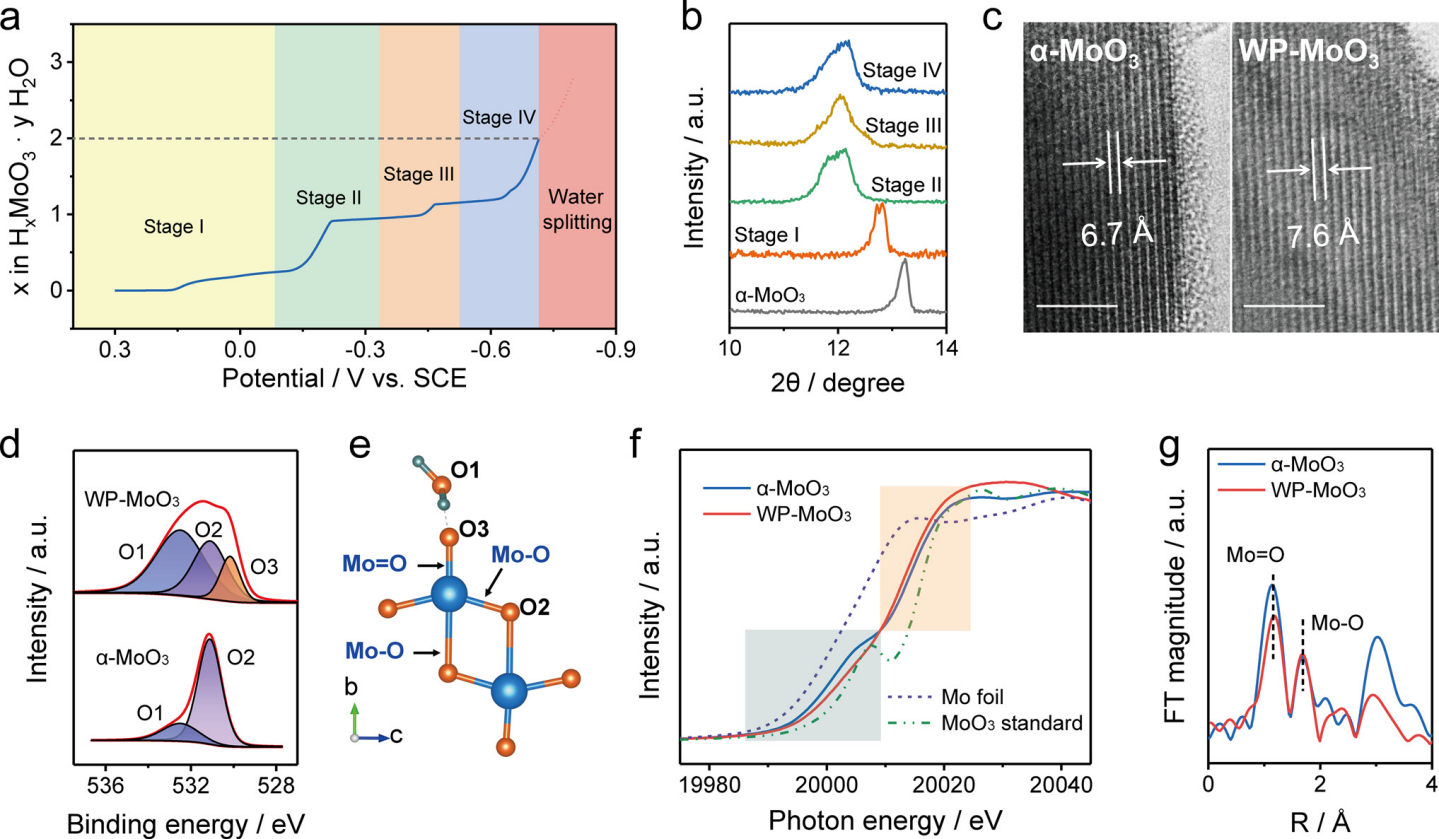
a) The intercalated proton amount as a function of potential during the water‐proton co‐intercalation process. b) XRD patterns of α‐MoO_3_ electrode at different intercalation stages. c) HRTEM images of α‐MoO_3_ and WP‐MoO_3_. Scale bars: 5 nm. d) O 1s XPS spectra of α‐MoO_3_ and WP‐MoO_3_. e) Illustration of different O atoms in WP‐MoO_3_. f) Normalized Mo K‐edge XANES spectra of MoO_3_ standard, Mo foil standard, α‐MoO_3_, and WP‐MoO_3_. g) Radial distribution functions of α‐MoO_3_ and WP‐MoO_3_ obtained from the *k* ^2^ *χ*(*k*) by Fourier transform.

α‐MoO_3_ nanoparticles (Figure S1) were first synthesized through a sol‐gel method. WP‐MoO_3_ electrode was obtained from α‐MoO_3_ electrode through a controllable and time‐efficient (≈6.1 min) electrochemical linear sweep voltammetry (LSV) method (Figure S2) in a three‐electrode cell with an electrolyte of 1 M H_2_SO_4_. Compared with α‐MoO_3_ electrode, WP‐MoO_3_ displays an apparent color change from dark grey to purple (Figure S3), which is attributed to the formation of Mo^4+^/Mo^5+^ (Figure S4).^[14]^ Almost no morphological variation was observed between α‐MoO_3_ and WP‐MoO_3_. The amount of intercalated H^+^ can be estimated by calculating the amount of charge transfer (Figure 2 a). The overall intercalation process presents four stages, referring to potential windows (vs. saturated calomel electrode (SCE)) of 0.3–−0.1 V (Stage I), −0.1–−0.34 V (Stage II), −0.34–−0.53 V (Stage III), and −0.53–−0.72 V (Stage IV). Approximately, the intercalated H^+^ numbers per MoO_3_ unit are 0.25, 0.75, 0.25, and 0.75 at Stage I, Stage II, Stage III, and Stage IV, respectively. X‐ray diffraction (XRD) spectra uncover that the peak position corresponding to the interlayer spacing of α‐MoO_3_ gradually shifts towards negative at Stage I and II and keeps almost unchanged at Stage III and IV (Figure 2 b & S5). This peak of WP‐MoO_3_ is located at 12.0°, indicating the interlayer distance expansion from 6.7 Å for α‐MoO_3_ to 7.6 Å (Figure S6). Besides, new peaks at 32.5°, 35.0°, and 37.1° are observed for WP‐MoO_3_, which indicates a monoclinic phase of proton‐intercalated MoO_3_.^[15]^ The widened interlayer distance of WP‐MoO_3_ was also evidenced by high‐resolution transmission electron microscopy (HRTEM) images, in which the interlayer spacings are determined to be 6.7 Å and 7.6 Å for MoO_3_ and WP‐MoO_3_, respectively (Figure 2 c & S7).

Figure 2 d compares the O 1s X‐ray photoelectron spectroscopy (XPS) spectra of MoO_3_ and WP‐MoO_3_ electrodes. Two peaks located at 532.6 eV and 531.2 eV are observed for α‐MoO_3_, which correspond to lattice O in MoO_3_ (denoted O2) and O in adsorbed H_2_O (denoted O1).^[16]^ Notably, WP‐MoO_3_ shows an O1 peak with the substantially enhanced intensity, verifying the intercalation of H_2_O into WP‐MoO_3_. Thermogravimetric analysis (TGA, Figure S8) results of both electrodes also suggest the intercalated H_2_O molecules in WP‐MoO_3_. More interestingly, an additional XPS peak at 530.2 eV (denoted O3) is observed for WP‐MoO_3_. This peak can be assigned to the terminal O of [MoO_6_] bilayers, which splits from O2 peak due to the formation of the hydrogen bond with the intercalated H_2_O/H_3_O^+^ (as illustrated in Figure 2 e). Furthermore, synchrotron‐based X‐ray absorption near‐edge spectra (XANES) measurements were performed to investigate the localized coordination environments of Mo sites in α‐MoO_3_ and WP‐MoO_3_. The Mo K‐edge XANES spectra of α‐MoO_3_ and WP‐MoO_3_, as well as standard Mo foil and MoO_3_ as references, are displayed in Figure 2 f. In comparison with α‐MoO_3_, the slightly negative‐shifted rising‐edge of WP‐MoO_3_ around 20 015 eV suggests the enriched electron densities around Mo sites.^[17]^ This result is consistent with the analysis of O K‐edge XANES spectra, which witness the decreased peak intensity of WP‐MoO_3_ at the energy region of 530–540 eV (Figure S9).^[18]^ In addition, the Mo pre‐edge of WP‐MoO_3_ around 20 007 eV, referring to the O 1s‐Mo 4d electron transfer, is obviously decreased compared with that of α‐MoO_3_, reflecting the interaction between the terminal O of [MoO_6_] bilayers and the intercalated species (i.e., H_2_O and H_3_O^+^).^[19]^ To acquire the detailed bonding and coordination information, corresponding R space curves after *k* ^2^[*χ*(*k*)]‐weighted Fourier transform of the extended X‐ray absorption fine structure (EXAFS) and quantitatively fitting spectra are presented in Figure 2 g & S10. Two representative peaks at 1.16 and 1.69 Å can be assigned to the scattering of Mo=O and Mo−O bonds in [MoO_6_] bilayers, respectively.^[20]^ These two peaks are also indicated in Figure 2 e. Both Mo=O and Mo−O bonds show negligible length difference between α‐MoO_3_ (1.72 & 1.96 Å) and WP‐MoO_3_ (1.73 & 1.97 Å), implying the water‐proton pre‐intercalation causes minor distortion of octahedron [MoO_6_] layers. Moreover, the coordination number of Mo−O (from 1.8 to 1.4) and Mo=O (from 2.0 to 1.4) decreases due to the water‐proton co‐intercalation, which again verifies the interaction between the terminal O of [MoO_6_] bilayers and the intercalated H_2_O/H_3_O^+^.

**Figure 3:**
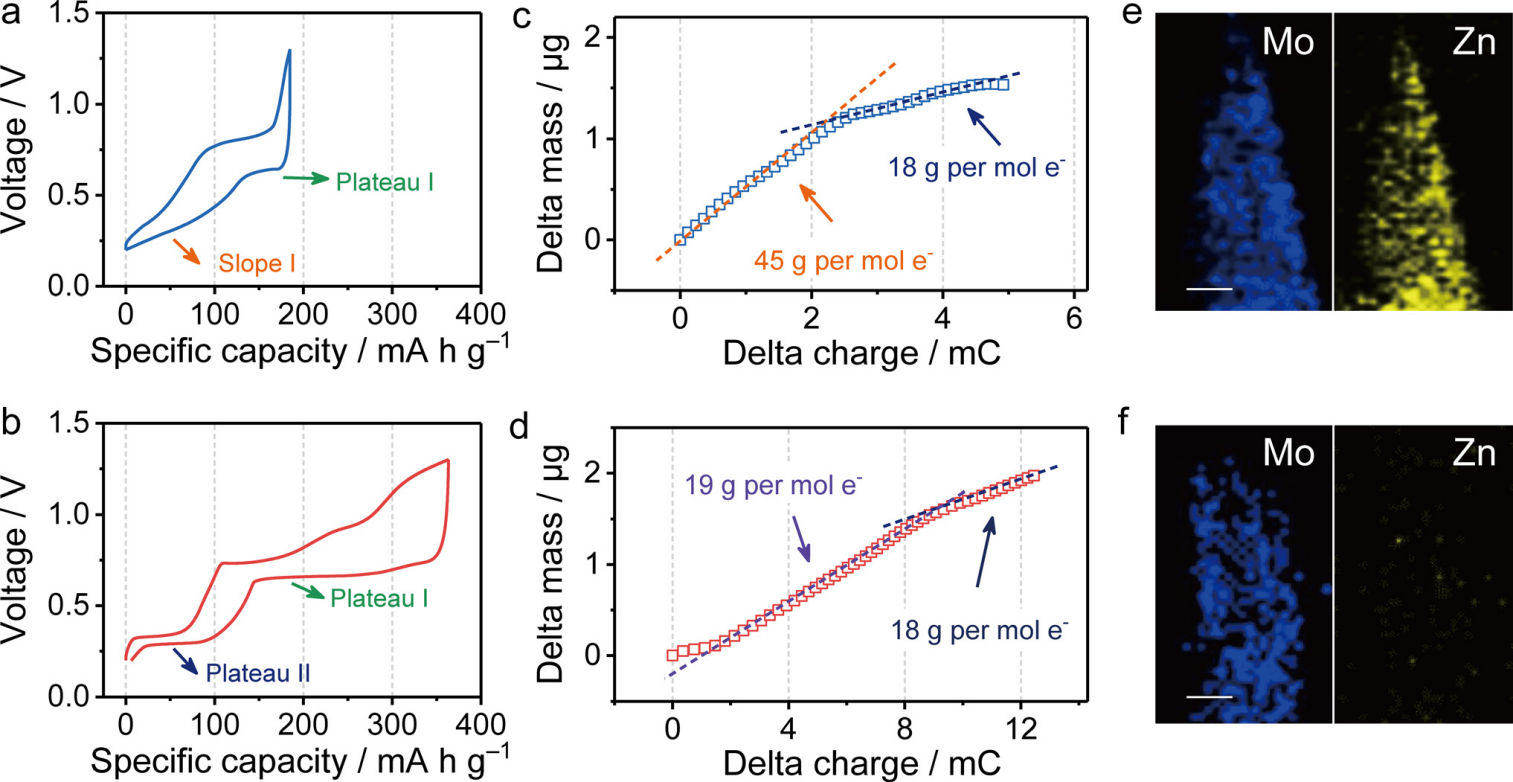
GCD profiles of a) α‐MoO_3_ and b) WP‐MoO_3_ electrodes at a current density of 0.4 A g^−1^. Electrode mass change versus charge during the discharge (ion‐intercalation) processes of c) α‐MoO_3_ and d) WP‐MoO_3_ electrodes. EDX elemental mapping of e) α‐MoO_3_ and f) WP‐MoO_3_ at the fully discharged state. Scale bars: 50 nm.

The ion‐intercalation behaviors of α‐MoO_3_ and WP‐MoO_3_ electrodes were explored in two‐electrode cells with Zn foil as the anode and 2 M ZnCl_2_ aqueous solution as the electrolyte. Figure 3 a and Figure 3 b present the galvanostatic charge/discharge (GCD) curves of α‐MoO_3_ and WP‐MoO_3_ electrodes after three‐cycle activation at 0.4 A g^−1^. Both electrodes show two mainly ion‐intercalation stages in their discharge curves, which agrees well with the cyclic voltammetry (CV) curves (Figure S11). In detail, α‐MoO_3_ electrode presents a discharge curve consisting of one plateau region (Plateau I, ≈0.62 V) and one slope region (Slope I, 0.25–0.35 V), while WP‐MoO_3_ electrode presents two plateaus around 0.68 V (Plateau I) and 0.30 V (Plateau II). Impressively, WP‐MoO_3_ electrode exhibits a high redox depth of 1.92 e^−^ per Mo atom, close to the full conversion of Mo^4+^/Mo^6+^. By contrast, a shallow redox depth of 0.99 e^−^ per Mo atom is achieved by α‐MoO_3_ electrode.

Electrochemical quartz crystal microbalance (EQCM) was employed to in‐operando monitor the mass evolution of MoO_3_ and WP‐MoO_3_ along with the continuous ion intercalation. In consistent with the discharge curves, the mass change of both electrodes during ion intercalation depicted two stages. At the first stage of α‐MoO_3_, the average weight increase calculated by the curve slope (Figure 3 c) is 45 g per mol charge, which suggests the co‐intercalation of Zn^2+^ and H_2_O into α‐MoO_3_ (Zn^2+^⋅1.5 H_2_O in average). Meanwhile, the average weight increase of α‐MoO_3_ at the second stage is 18 g per mol charge, close to the weight of H_3_O^+^ (19 g per mol charge). Clearly, Zn^2+^/H_2_O co‐intercalation plays the dominant role in the charge‐storage mechanism of α‐MoO_3_. By contrast, WP‐MoO_3_ depicts the weight increase of 19 and 18 g per mol charge at the two stages (Figure 3 d), verifying the selective H_3_O^+^‐intercalation chemistry of WP‐MoO_3_ in 2 M ZnCl_2_ electrolyte. This result means although Zn^2+^ is the mainly charge carrier in the electrolyte, the charge carrier only consists of H_3_O^+^ in WP‐ MoO_3_ electrode, which comes from the pre‐stored H^+^ in WP‐MoO_3_ and the slight hydrolysis of ZnCl_2_ in the electrolyte. However, the local Zn^2+^ concentration near the surface of WP‐MoO_3_ should be increased during discharging, because Zn^2+^ tends to migrate to the Helmholtz layer of WP‐MoO_3_ due to the electrostatic interaction.

The interesting H_3_O^+^‐intercalation behavior of WP‐MoO_3_ is further supported by the Energy‐dispersive X‐ray spectroscopy (EDX) elemental mapping analysis. The fully discharged α‐MoO_3_ presents the even distribution of Mo and Zn over the sample (Figure 3 e & S12), while Zn distribution is barely observed in WP‐MoO_3_ (Figure 3 f & S12). The Zn/Mo atomic ratio of the discharged WP‐MoO_3_ is calculated to be 0.02, which contrasts with the high Zn/Mo atomic ratio of the discharged α‐MoO_3_ (0.48). Moreover, the discharged α‐MoO_3_ and WP‐MoO_3_ were annealed in air at 500 °C and subjected for XRD measurements (Figure S13). Peaks refer to ZnMoO_3_ are only detected for the discharged α‐MoO_3_, rather than for the discharged WP‐MoO_3_. All these results identify the successful modulation of intercalating charge carriers for α‐MoO_3_, which brings the obtained WP‐MoO_3_ with exceptional selective H_3_O^+^‐intercalation chemistry in a neutral electrolyte.

**Figure 4:**
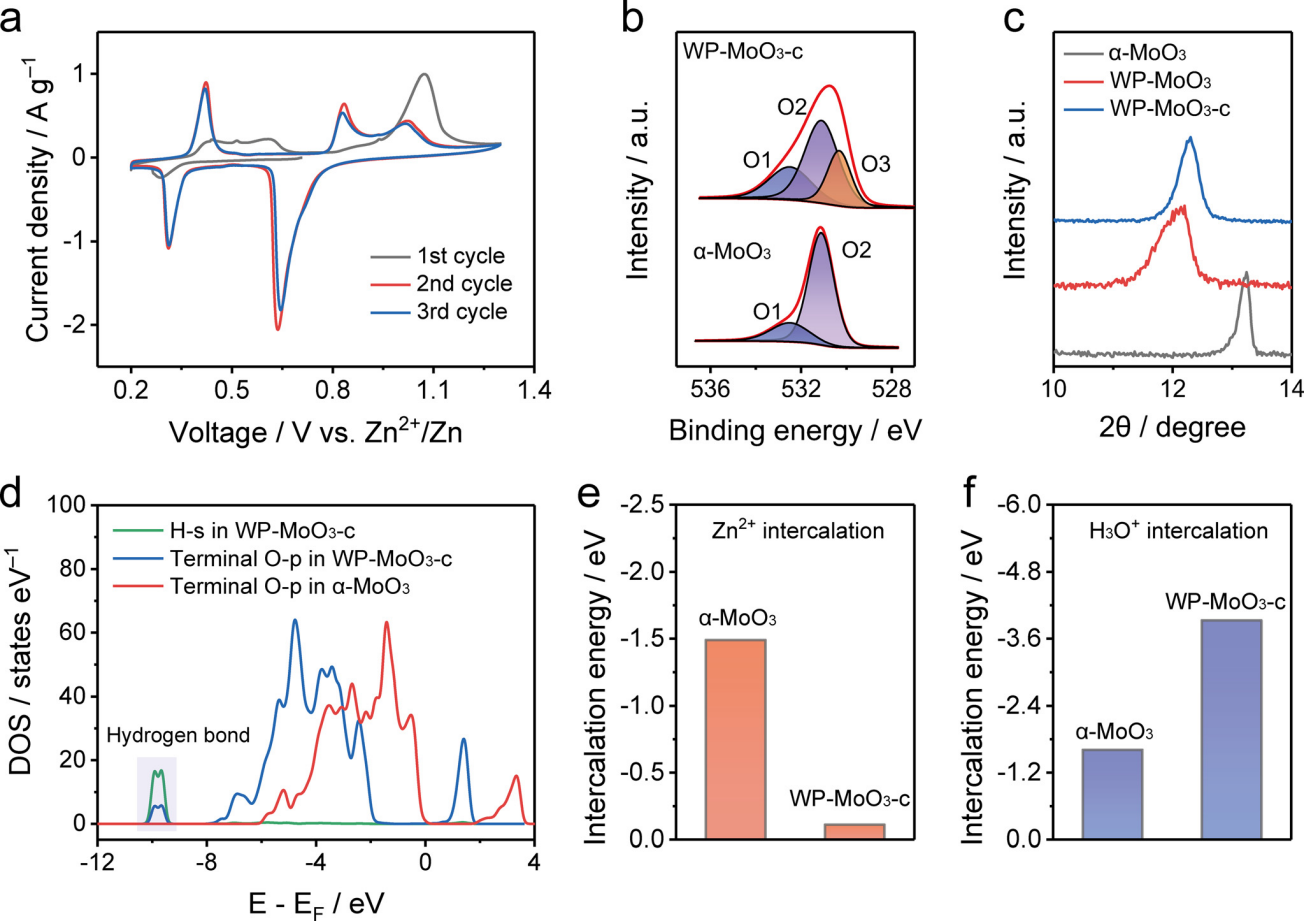
a) Initial three CV cycles of WP‐MoO_3_ at 0.7 mV s^−1^. b) O 1s XPS spectra of α‐MoO_3_ and WP‐MoO_3_‐c. c) XRD patterns of α‐MoO_3_, WP‐MoO_3_, and WP‐MoO_3_‐c. d) Simulated PDOS of α‐MoO_3_ and WP‐MoO_3_‐c. Calculated e) Zn^2+^‐intercalation energy and f) H_3_O^+^‐intercalation energy for α‐MoO_3_ and WP‐MoO_3_‐c.

To understand the origin of the selective H_3_O^+^‐intercalation behavior, the first three CV cycles of WP‐MoO_3_ electrode were recorded as shown in Figure 4 a. In the initial cycle, only a small cathodic peak is observed during the discharge process, whereas the charge process displays four anodic peaks corresponding to the extraction of the pre‐intercalated H_3_O^+^ and H_2_O. Afterwards, WP‐MoO_3_ electrode presents almost the identical 2^nd^ and 3^rd^ cycles (Figure S14), which include three anodic peaks and three cathodic peaks. The cathodic peaks and anodic peaks can be assigned to the H_3_O^+^ intercalation and de‐intercalation, respectively. Based on the CV curves, the charge transfer number of the first two pairs of redox peaks is calculated to be 1.495 (close to 1.5), implying the conversion between Mo^6+^ and Mo^5+^/Mo^4+^ (1:1). Meanwhile, the charge transfer of the third pair of redox peaks is 0.498 (close to 0.5), which refers to the conversion between Mo^5+^/Mo^4+^ (1:1) and Mo^4+^. In this regard, the structure of WP‐MoO_3_ after the first cycle (denoted WP‐MoO_3_‐c) is of significance to induce the selective H_3_O^+^ intercalation of WP‐MoO_3_ electrode. The Mo 3d XPS spectrum of WP‐MoO_3_‐c uncovers the existence of Mo^5+^ in WP‐MoO_3_‐c (Figure S15), indicating that H^+^ ions were not fully extracted from the lattice. This result is further supported by the observation of O3 peak in the O 1s XPS of WP‐MoO_3_‐c (Figure 4 b). The partial extraction of H_2_O was verified by the larger O1 peak intensity of WP‐MoO_3_‐c than that of α‐MoO_3_, as well as the TGA analysis (Figure S16). Moreover, the interlayer distance of WP‐MoO_3_‐c identified by the XRD peak position only slightly decreased from 7.6 Å for WP‐MoO_3_ to 7.4 Å, which remains to be significantly larger than that of α‐MoO_3_ (6.7 Å) (Figure 4 c & S17).

Based on the quantitive analysis of XPS results, the structures of α‐MoO_3_ and WP‐MoO_3_‐c (Figure S18) were simulated with the density functional theory (DFT) method. In WP‐MoO_3_‐c, the bonding interaction between the residual intercalant (i.e., H_2_O and H_3_O^+^) and the terminal O of [MoO_6_] bilayers is proved by the formant arise at −10 eV in projected density of states (PDOS) of terminal O‐p orbital with H‐s orbital (Figure 4 d). This bonding interaction greatly influence the electron density of terminal O atoms, which agrees well with the O 1s XPS results of our samples. Additionally, WP‐MoO_3_ has a band gap of 0.04 eV, which is remarkably smaller than that of α‐MoO_3_ (2.18 eV). The Fermi level of WP‐MoO_3_ also shifts to the conduction band, favoring the excitation of charge carriers to the conduction band and thus the improved electronic conductivity (Figure S19).^[21]^

**Figure 5:**
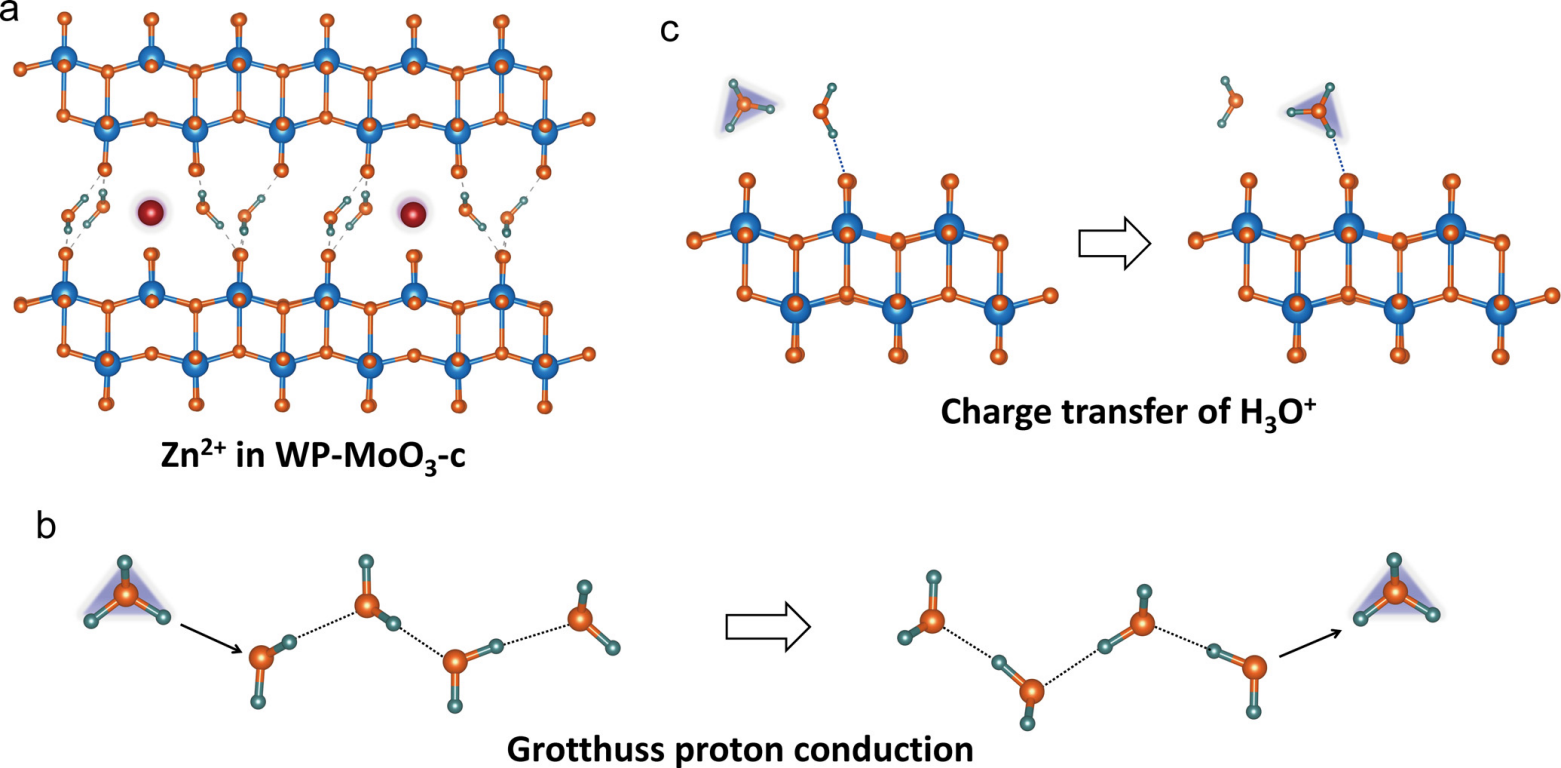
a) Schematic illustration of Zn^2+^ inserted WP‐MoO_3_‐c. b) Schematic illustration of the Grotthuss proton‐conduction mechanism. Protons are transported by rearranging bonds along a water chain. c) Schematic illustration of the charge transfer of H_3_O^+^ via Grotthuss mechanism.

Subsequently, the Zn^2+^‐intercalation energy (Figure 4 e) and H_3_O^+^‐intercalation energy (Figure 4 f) were calculated for α‐MoO_3_ and WP‐MoO_3_‐c. As expected, a substantially enlarged Zn‐intercalation energy is uncovered for WP‐MoO_3_‐c (−0.11 eV) in comparison to α‐MoO_3_ (−1.49 eV), while the H_3_O‐intercalation energy of WP‐MoO_3_‐c (−3.93 eV) is notably lower than that of α‐MoO_3_ (−1.61 eV). These results confirm the thermodynamically preferable H_3_O^+^ intercalation into WP‐MoO_3_‐c. Besides, the residual H_2_O and H_3_O^+^ located in the interlayer space and bonded with terminal O atoms of [MoO_6_] bilayers block the Zn^2+^‐diffusion pathways in WP‐MoO_3_‐c (Figure 5 a) and hinder the charge transfer through the interaction with [MoO_6_] bilayers (Figure S20 & 21). In the case of H_3_O^+^ intercalation into WP‐MoO_3_‐c, charge carrier diffusion can be efficiently achieved through a well‐established Grotthuss mechanism (Figure 5 b), in which protons can be fast transported by “jumping” through water molecules.^[22]^ Proton conductivities measurement (Figure S22) shows that WP‐MoO_3_‐c owns a proton conductivity value of 4.3×10^−3^ S cm^−1^ at 318 K and 100 % humidity, which is much higher than α‐MoO_3_ (6.2×10^−5^ S cm^−1^). Moreover, WP‐MoO_3_‐c also shows an activation energy (*E*_a_) of 0.28 eV, which suggests the Grotthuss conduction mechanism (*E*_a_<0.4 eV).^[23]^ H_2_O molecules between the interlayers serve as the proton transport intermedia, providing a hydrogen‐bonding network for the high‐kinetics charge carrier diffusion.^[24]^ Moreover, the charge transfer from H_3_O^+^ to WP‐MoO_3_‐c can be also through Grotthuss mechanism without breaking the hydrogen bonding interaction between H_2_O and the terminal O of [MoO_6_] bilayers (Figure 5 c). Thereby, the unique van der Waals structure of WP‐MoO_3_‐c favors the selective H_3_O^+^‐intercalation behavior both thermodynamically and kinetically.

**Figure 6:**
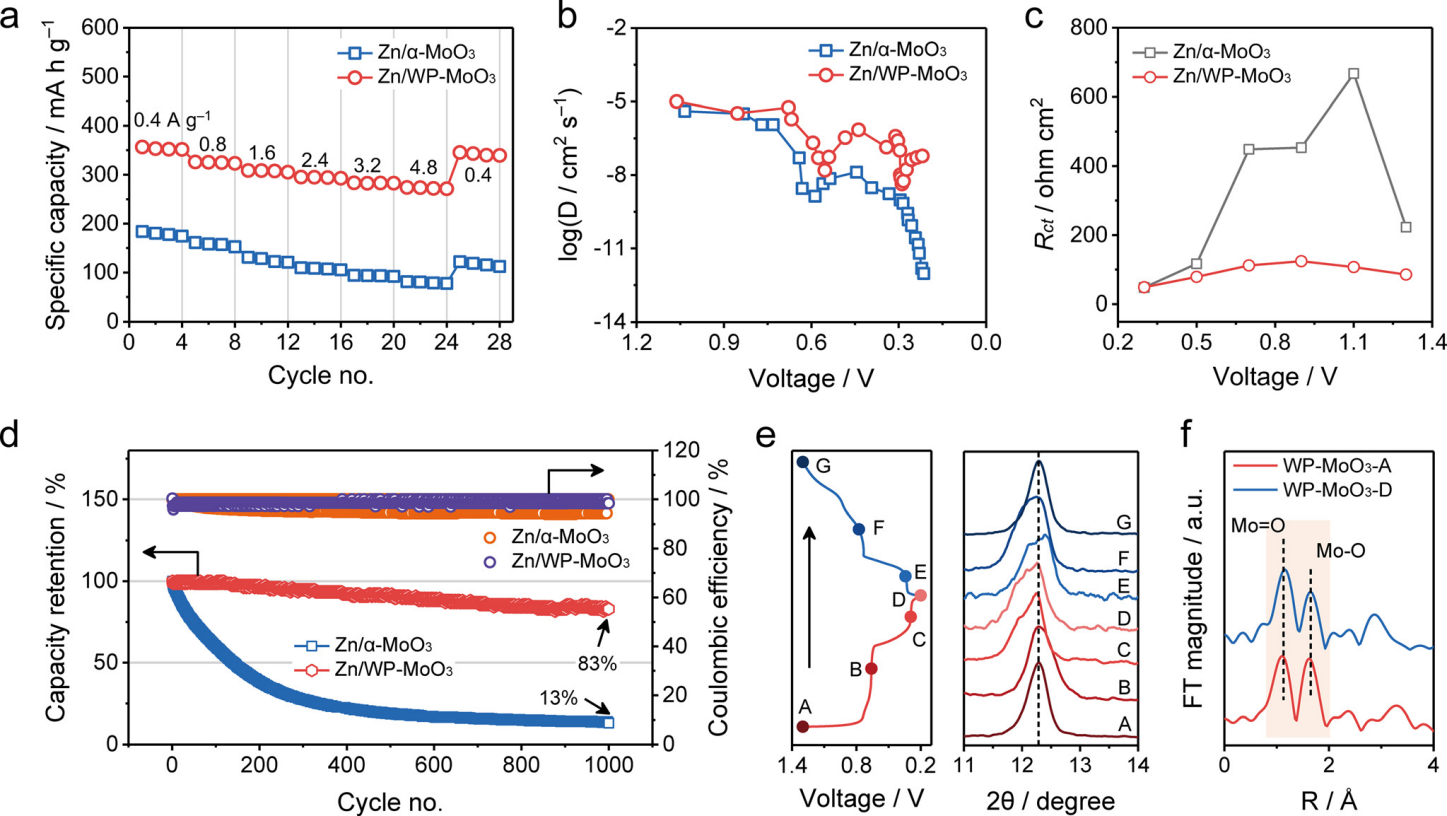
a) Rate performance, b) ion diffusion coefficient as a function of the voltage during the discharge, c) *R*_ct_ at different voltages, and d) cycling performance at 3.2 A g^−1^ of Zn/α‐MoO_3_ and Zn/WP‐MoO_3_ devices. e) Ex situ XRD of WP‐MoO_3_ during one discharge/charge cycle of Zn/WP‐MoO_3_. f) Radial distribution functions obtained from the *k* ^2^ *χ*(*k*) by Fourier transform of XANES of WP‐MoO_3_ cathode at the charged (WP‐MoO_3_‐A) and discharged (WP‐MoO_3_‐D) states.

The selective H_3_O^+^ intercalation of WP‐MoO_3_ electrode motivated us to assess the electrochemical performance of ZMB devices assembled by coupling Zn anodes with WP‐MoO_3_ cathodes (denoted Zn/WP‐MoO_3_). ZMB devices based on Zn^2+^‐intercalation α‐MoO_3_ cathodes were also constructed for comparison (denoted Zn/α‐MoO_3_). GCD curves at various current densities were collected to evaluate both devices (Figure S23). As shown in Figure 6 a, Zn/α‐MoO_3_ only exhibits a specific capacity of 184.0 mA h g^−1^ at 0.4 A g^−1^ (based on the cathode) and a capacity retention of 42.2 % at 4.8 A g^−1^. By contrast, Zn/WP‐MoO_3_ delivers a much larger specific capacity (356.8 mA h g^−1^ at 0.4 A g^−1^) and a greatly improved rate capability (77.5 % from 0.4 to 4.8 A g^−1^). When the current density returns to 0.4 A g^−1^ after the rate tests, Zn/WP‐MoO_3_ can still show a specific capacity of 345.6 mA h g^−1^. The influence of different amounts of intercalated H^+^ (from 0.25 to 2.0 per MoO_3_) on the electrochemical performance was also investigated by preparing a series of WP‐MoO_3_ electrodes with different cut‐off potentials (Figure S24). In brief, the larger amount of intercalated H^+^ results in the higher specific capacity of the obtained Zn/WP‐MoO_3_ devices. Importantly, Zn/WP‐MoO_3_ delivers the maximum energy density of 198.0 Wh kg^−1^ (based on the cathode) at a power density of 0.28 kW kg^−1^, as well as the peak power density of 6.7 kW kg^−1^ at a high energy density of 104.5 Wh kg^−1^. These energy and power densities significantly outperform those of the recently reported ZMBs based on cathodes like MnO_2_,^[3c]^ ZnMn_2_O_4_,^[25]^ VS_2_,^[26]^ ZnHCF,^[27]^ and pyrene‐4,5,9,10‐tetraone^[28]^ (Figure S25). It should be noticed that the energy contribution of plateau II (≈0.30 V) is only about 16.8 % in Zn/WP‐MoO_3_ (Figure S26), while most of the energy is contributed by plateau I (≈0.68 V). The outstanding performance of Zn/WP‐MoO_3_ originates from H_3_O^+^ charge carriers, which allow the high‐kinetics diffusion and the efficient charge transfer. Figure 6 b displays the calculated charge carrier diffusion coefficients of Zn/α‐MoO_3_ and Zn/WP‐MoO_3_ devices based on a galvanostatic intermittent titration technique (GITT, Figure S27). Both devices exhibit low charge carrier diffusion coefficients at voltages associated with ion‐intercalation stages. As expected, Zn/WP‐MoO_3_ with H_3_O^+^ charge carriers achieves high diffusion coefficients of 1.6×10^−8^ cm^2^ s^−1^ at 0.57 V and 4.4×10^−9^ cm^2^ s^−1^ at 0.29 V, which are several orders of magnitude higher than those of Zn/α‐MoO_3_ with Zn^2+^ as the main charge carrier (1.2×10^−9^ cm^2^ s^−1^ at 0.58 V and 1.3×10^−12^ cm^2^ s^−1^ at 0.22 V). In addition, the low charge transfer resistance (*R*_ct_) of Zn/WP‐MoO_3_ derived from electrochemical impedance spectroscopy (EIS, Figure S28) reflects the efficient charge transfer enabled by H_3_O^+^ charge carriers. As shown in Figure 6 c, *R*_ct_ of Zn/WP‐MoO_3_ ranges from 48.9 to 124.5 Ω, which contrasts with that of Zn/α‐MoO_3_ (48.1 to 667.3 Ω).

The cycling stability of Zn/α‐MoO_3_ and Zn/WP‐MoO_3_ was evaluated at a current density of 3.2 A g^−1^. Zn/WP‐MoO_3_ presents impressive coulombic efficiencies of nearly 100 %, which indicates its high charge/discharge reversibility. In contrast with the fast capacity decay of Zn/α‐MoO_3_ (13 % capacity retention after 1000 cycles), Zn/WP‐MoO_3_ can maintain 83 % of the initial capacity after 1000 cycles (Figure 6 d). The outstanding cycling performance of Zn/WP‐MoO_3_ is assigned to the negligible structure distortion of WP‐MoO_3_ during the repeated H_3_O^+^ intercalation/extraction. In the ex‐situ XRD tests of WP‐MoO_3_ cathode during one discharge/charge cycle of Zn/WP‐MoO_3_, the peak position located at 12.3° of WP‐MoO_3_ only experiences slight shift, indicating the little volume expansion/shrinkage of WP‐MoO_3_ in direction which is perpendicular to interlayer (Figure 6 e). Moreover, Mo‐K edge XANES (Figure S29) and corresponding Fourier transforms of the Mo K‐edge *k* ^2^ *χ*(*k*) spectra (Figure 6 f) confirm that both Mo=O and Mo−O bonds of WP‐MoO_3_ cathode have no change in bonding length at the fully charged and discharged stages. All these results imply that the selective H_3_O^+^‐intercalation chemistry is able to empower electrodes with large specific capacity, high charge‐storage kinetics and long cycle life.

## Conclusion

In summary, we have uncovered an exceptional selective H_3_O^+^‐intercalation chemistry for WP‐MoO_3_ electrode in a neutral ZnCl_2_ electrolyte. The interesting charge‐carrier‐selection behavior of WP‐MoO_3_ originated from the interlayer species (i.e., H_2_O, H_3_O^+^) of WP‐MoO_3_, which allowed the fast‐kinetics transport and charge transfer of H_3_O^+^ while blocking Zn^2+^ intercalation. This study provides a novel charge‐carrier‐modulation concept through the strategic van der Waals structure engineering, which opens a promising prospect for developing high‐kinetics and long‐life battery technologies.

## Conflict of interest

The authors declare no conflict of interest.

## Supporting information

As a service to our authors and readers, this journal provides supporting information supplied by the authors. Such materials are peer reviewed and may be re‐organized for online delivery, but are not copy‐edited or typeset. Technical support issues arising from supporting information (other than missing files) should be addressed to the authors.

Supplementary
