# Supplementary material for: Interlayer Engineering of α‐MoO3 Modulates Selective Hydronium Intercalation in Neutral Aqueous Electrolyte
Source: Angew Chem Int Ed Engl. 2020 Nov 9;60(2):896–903. doi: 10.1002/anie.202010073 (PMC7839748; doi:10.1002/anie.202010073)
Supplement: Supplementary file 1 — Supplementary [file ANIE-60-896-s001.pdf]

Angewandte Chemie
Eine Zeitschrift der Gesellschaft Deutscher Chemiker

Supporting Information

Interlayer Engineering of α-MoO_3 Modulates Selective Hydronium Intercalation in Neutral Aqueous Electrolyte

Haozhe Zhang, Weixing Wu, Qiyu Liu, Fan Yang, Xin Shi, Xiaoqing Liu, Minghao Yu, and Xihong Lu**

anie_202010073_sm_miscellaneous_information.pdf

Experimental Procedures

Preparation of α-MoO_3 and WP-MoO_3 electrodes. All the chemicals were of analytical grade and used directly without any purification. α-MoO_3 nanoparticles were first prepared through a sol-gel reaction. 1 g of $(NH_4)_6Mo_7O_{24}$ and 1.7 g of citric acid monohydrate were dissolved into 10 mL of ethylene glycol at 80 °C. 30% ammonia (about 5 mL) was dropwise added into the solution to neutralize the solution (pH = 7), forming a transparent colorless solution. Afterwards, the solution in a sealed glass bottle was heated by an oil bath, and stirred at 80 °C for 3 hours and at 120 °C for another 6 hours. After cooling down to room temperature, the obtained gel-like sample was transferred into a nickel crucible and annealed under air at 500 °C for 1 hour to obtain α-MoO_3 nanoparticles.

α-MoO_3 slurry was prepared by mixing α-MoO_3 powder with carbon black and polyvinylidene fluoride (PVDF) binder with a weight ratio of 8: 1: 1 in N-methyl-2-pyrrolidinone. α-MoO_3 electrode was fabricated by casting the slurry on carbon fiber paper (Shanghai Hesen Electric Company) substrate through a conventional blade-coating method. Finally, α-MoO_3 electrode was dried overnight in a vacuum oven at 100 °C. The areal mass loading of α-MoO_3 is 2.5 mg cm^{-2}. While preparing TGA samples, the substrate was replaced by Ti foil.

WP-MoO_3 electrode was obtained by a facile electrochemical treatment of α-MoO_3 electrode in a typical three-electrode system with a 1 M H_2SO_4 aqueous electrolyte. α-MoO_3 electrode with a size of 1×1 cm^2 was used as the working electrode, while an over-capacity graphite rod and a saturated calomel electrode (SCE) were used as the counter electrode and the reference electrode, respectively. A linear sweep voltammetry (LSV) method was conducted from the open circuit potential (0.3 V *vs.* SCE) to −0.8 V *vs.* SCE at a scan rate of 3 mV s^{-1}. The electrochemical treatment only took 6.1 min. WP-MoO_3 electrodes with different amounts of intercalated H^+ were prepared by the same electrochemical activation method, but with different cut-off potentials. The cut-off potentials are chosen according to different intercalation stages, and the electrodes are denoted as WP-MoO_3-I (−0.1 V *vs.* SCE, 0.25 H^+ per MoO_3), WP-MoO_3-II (−0.34 V *vs.* SCE, 1.0 H^+ per MoO_3), WP-MoO_3-III (−0.53 V *vs.* SCE, 1.25 H^+ per MoO_3), and WP-MoO_3-IV (−0.8 V *vs.* SCE, 2.0 H^+ per MoO_3). The optimal WP-MoO_3 electrode (WP-MoO_3-IV) was prepared with a cut-off potential of −0.8 V *vs.* SCE, which is denoted as WP-MoO_3 in the main text.

Characterizations. The microstructures and chemical compositions of the electrode materials were analyzed using transmission electron microscopy (TEM, FEI Tecnai G^2 F30), field-emission scanning electron microscopy (FE-SEM, JSM-6330F), X-ray diffraction (XRD, D-MAX 2200 VPC, RIGAKU), and X-ray photoelectron spectroscopy (XPS, NEXSA, Thermo VG). XPS was performed by using Al K Alpha source gun (h*v*=1486.6 eV), an energy step size of 0.05 eV, and a pass energy of 40 eV. Thermogravimetric analysis (TGA) was conducted using a TG209F1 libra (Netzsch, Germany) analyzer at a scan rate of 10 °C min^{-1} from 25 to 500 °C in N_2.

X-ray absorption measurements were carried out at the BL14W1 beamline of the Shanghai Synchrotron Radiation Facility (SSRF). The X-ray absorption near-edge structure (XANES) measurements at the Mo *K*-edge was performed at Wiggler beamline 17C using a transmission cell. A double crystal Si (111) monochromator was used with an energy resolution ΔE/E better than 2×10^{-4}. The data is collected in fluorescence yield (TFY). MoO_3 standard and Mo foil standard were used as reference samples, and the photon energy of

α-MoO_3 and WP-MoO_3 was calibrated by the Mo foil standard. For the extended X-ray absorption fine structure (EXAFS), the Fourier-transformed data in R space were analyzed by applying the 1st shell approximation or metallic Mo model for the Mo-Mo shell, respectively. The passive electron factor (S_0^2) in the fitting was determined by fitting the experimental spectra of Mo foil and fixing the Mo-Mo coordination number to be 8 and 6. Then, S_0^2 was fixed for further analysis of the measured samples. The parameters describing the local structure environment, including coordination number (*N*), bond distance (R), and Debye-Waller (DW) factor around the studied atoms, were allowed to vary during the fitting process. The O *K*-edge XANES spectra were collected at the photoemission end-station at beamline BL10B in the National Synchrotron Radiation Laboratory (NSRL) in Hefei, China.

Regarding the *ex-situ* XRD and XANES tests, the electrodes were first stabilized at specific potentials for 10 min and then washed with distilled water to avoid residual electrolyte. Before characterization, the electrodes were dried overnight in a vacuum oven at 100 °C.

Electrochemical measurements. All the electrochemical tests were performed in a two-electrode system with a 2 M $ZnCl_2$ aqueous electrolyte by using an electrochemical workstation (Princeton, PARSTAT MC). α-MoO_3 electrode or WP-MoO_3 electrode with a size of 1×1 cm^2 was used as the cathode, and a piece of Zn foil was used as the anode. Cyclic voltammetry (CV) and galvanostatic charge-discharge (GCD) tests were conducted with a voltage window of 0.2-1.3 V. For energy contribution test of two plateaus, the voltage window was set as 0.4-1.3 V. Electrochemical impedance spectroscopy (EIS) measurements were performed in a frequency range of 0.01 Hz ~ 100 kHz.

The electrochemical properties including specific capacity (C_m, mA h g^{-1}), mass energy density *E* (W h kg^{-1}) and mass power density *P* (kW kg^{-1}) were calculated from GCD curves of the battery based on equation (1-3), where *I* stands for the current (A), Δt stands for the discharge time (h), *S* stands for the test area (cm^{-2}), *m* stands for the mass loading (mg cm^{-2}) and V stands for the voltage (V).

$$C_m = \frac{10^6 \times I \cdot \Delta t}{m \times S} \quad (1)$$

$$E = \int C_m dV \quad (2)$$

$$P = \frac{E}{1000 \times \Delta t} \quad (3)$$

galvanostatic intermittent titration technique (GITT) tests were performed with a current pulse of 800 mA g^{-1} for 60 s and a rest interval of 30 min. The ion diffusion coefficient (*D*) was calculated based on equation (4), where *I* stands for the current (A), V_m stands for the molar volume of the cathode material (cm^3 mol^{-1}), Z_A stands for the charge number, *F* stands for the Faraday's constant (96485 C mol^{-1}), *S* stands for the contact area of electrode and electrolyte (cm^2). $dE/d\delta$ and $dE/d\sqrt{t}$ can be simplified into ΔE_s (V) and ΔE_t (V), respectively. ΔE_s is the steady-state voltage change due to the current pulse and ΔE_t is the voltage change during the constant current pulse, eliminating the iR drop.

$$D = \frac{4}{\pi}\left(I\frac{V_m}{Z_A F S}\right)^2\left(\frac{dE/d\delta}{dE/d\sqrt{t}}\right)^2 \quad (4)$$

EQCM measurements. Electrochemical quartz crystal microbalance (EQCM) measurements were conducted by using a QCM 200 (Stanford Research Systems) and a BioLogic potentiostat. EQCM curves were recorded during the discharge process by CV scan at 1 mV s^{-1}. The mass change (Δm, g) of the electrode can be calculated by equation (5), where ρ_q and μ_q stands for the density (2.648 g cm^{-3}) and shear modulus (2.947 × 1011 g cm^{-1} s^{-2}) of quartz, respectively. f_0 (Hz) is the fundamental resonance frequency of quartz. Δm (g) and Δf (Hz) are the mass change and frequency change, respectively. C_f (14.6 ng/Hz) is the sensitivity factor calculated by the relation based on frequency and mass change between the quartz crystal before and after coating. The molar weight of charge carrier (M_w) can be calculated according to equation (6), where F stands for the Faraday constant (96485 C mol^{-1}), n stands for the valence number of the ion, and ΔQ (C) stands for the charges passed through during the electrochemical process.

$$\Delta m = \frac{\sqrt{\rho_q \mu_q}}{2f_0} * \Delta f = -C_f * \Delta f \qquad (5)$$

$$M_w = \frac{nf\Delta m}{\Delta Q} \qquad (6)$$

Proton conductivity tests. α-MoO_3 powder was mixed with PVDF (10% mass ratio) and compressed into a pellet on a stainless steel substrate with a diameter of 1.3 cm and a thickness of 0.2 mm. The pellet was activated in 1 M H_2SO_4 aqueous electrolyte to obtain WP-MoO_3 pellet. WP-MoO_3-c pellet was obtained from WP-MoO_3 pellet after the first cycle of cyclic voltammetry scan. The pellets were assembled into coin cells, in which wet cotton balls were placed to maintain the 100% humidity during tests. During the EIS test, the oscillation amplitude was set as 20 mV and the frequencies range from 1 MHz to 0.1 Hz. We adopted an equivalent circuit (shown below) to fit the data in Z-plot software.

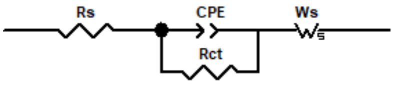

The proton conductivity can be determined by equation (7), where σ represents the proton conductivity (S cm^{-1}), R_b represents the bulk resistance (Ω), L is the sample thickness (cm), and S refers to the sample area (cm^2).

$$\sigma = L/R_b S \qquad (7)$$

The activation energy (E_a) can be determined according to equation (8), where E_a represents the activation energy for proton conduction (eV), T refers to the temperature (K), A refers to the pre-exponential factor, and K_b represents the Boltzmann constant (8.6×10^{-5} eV K^{-1}).

$$\ln(\sigma T) = \ln A - E_a/K_b T \qquad (8)$$

DFT calculations. All the theoretical calculations were carried out by the CASTEP module implemented in the Materials Studio 8.0 software (Accelrys Software, Inc.), which based on the plane wave pseudo-potential approach. The geometry optimization utilized the generalized gradient approximation (GGA) and the Perdew-Burke-Ernzerh (PBE) of exchange correlation function. The Grimme method was applied for the DFT-D correction. Cut-off energy was set as 450 eV. Ultrasoft pseudopotential were adopted to describe the core

electrons. For the geometric optimization, the Brillouin zone was sampled with a 2×2×2 k-point mesh. A Monkhorst–Pack scheme with 4×4×4 k-point grid meshes was employed for the electronic structure density of states calculations. The convergence criteria of energy tolerance, maximum displacement tolerance, maximum force tolerance, max stress tolerance and self-consistent field tolerance for geometry optimization were set as 1.0×10^{-5} eV atom^{-1}, 1.0×10^{-3} Å, 0.03 eV Å^{-1}, 0.05 GPa and 1.0×10^{-6} eV atom^{-1}, respectively. The intercalation energies ($E_{\text{intercalation}}$) were identified by the equation (9). For Zn^{2+}, an extra $E_{\text{hydration}}$ should be subtracted. The $E_{\text{hydration}}$ was calculated by equation (10). $E_{\text{ion+bulk}}$ is the energy of crystal with intercalated ions. E_{bulk} and E_{ion} are the energies of separated crystal and intercalated ions.

$$E_{\text{intercalation}} = E_{\text{bulk+ion}} - E_{\text{bulk}} - E_{\text{ion}} \qquad (9)$$

$$E_{\text{hydration}} = E_{\text{zinc hydrate}} - E_{\text{zinc ion}} - E_{\text{water}} \qquad (10)$$

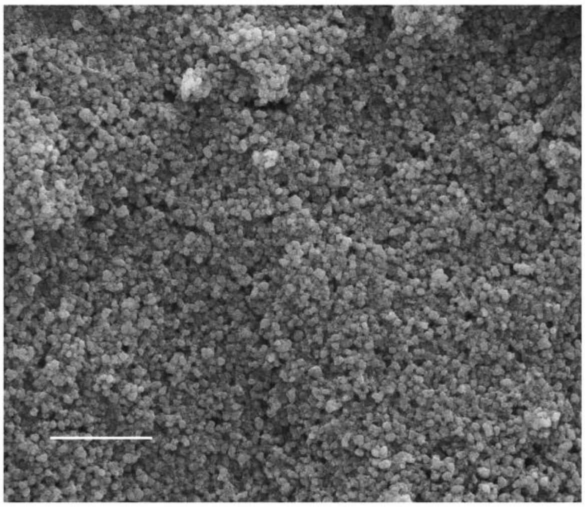

Figure S1. SEM image of α-MoO_3 nanoparticles. Scale bar:10 μm.

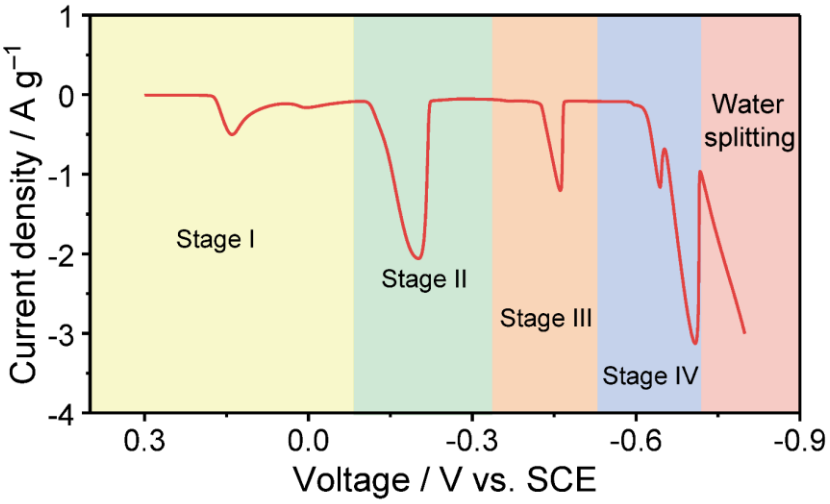

Figure S2. LSV curve of the water-proton co-intercalation process. The scan rate is 3 mV s^{-1}. Stage I-IV correspond to the potential ranges (*vs.* SCE) of 0.3 ~ −0.1 V, −0.1 ~ −0.34 V, −0.34 ~ −0.53 V and −0.53 ~ −0.72 V, respectively.

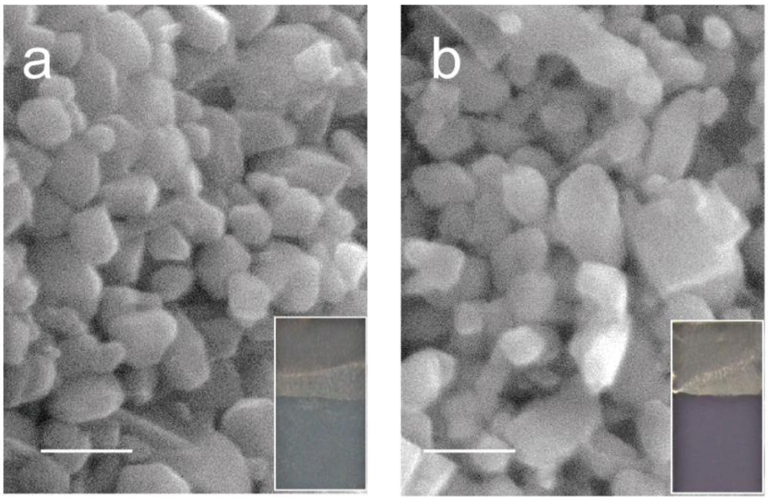

Figure S3. SEM images of α-MoO_3 and WP-MoO_3 electrodes. The SEM images and photographs (insets, on Ti foil) of (a) α-MoO_3 and (b) WP-MoO_3 electrodes. Scale bars: 500 nm.

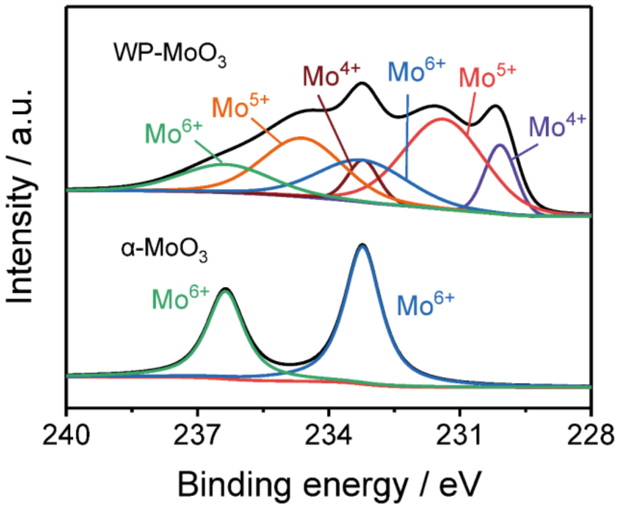

Figure S4. XPS Mo 3d spectra of α-MoO_3 and WP-MoO_3. α-MoO_3 exhibits only a pair of doublets at 233.2 eV and 236.4 eV corresponding to the peaks of Mo^{6+}. Meanwhile, WP-MoO_3 exhibits three sets of doublets, corresponding to Mo^{4+}, Mo^{5+}, and Mo^{6+}.

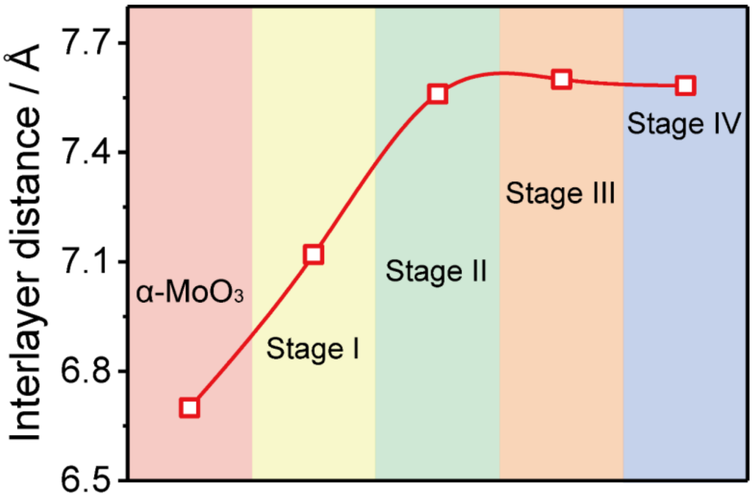

Figure S5. Calculated interlayer distance of WP-MoO_3 at different co-intercalation stages. The interlayer distance increased at stage I and II and kept almost unchanged at stage III and IV.

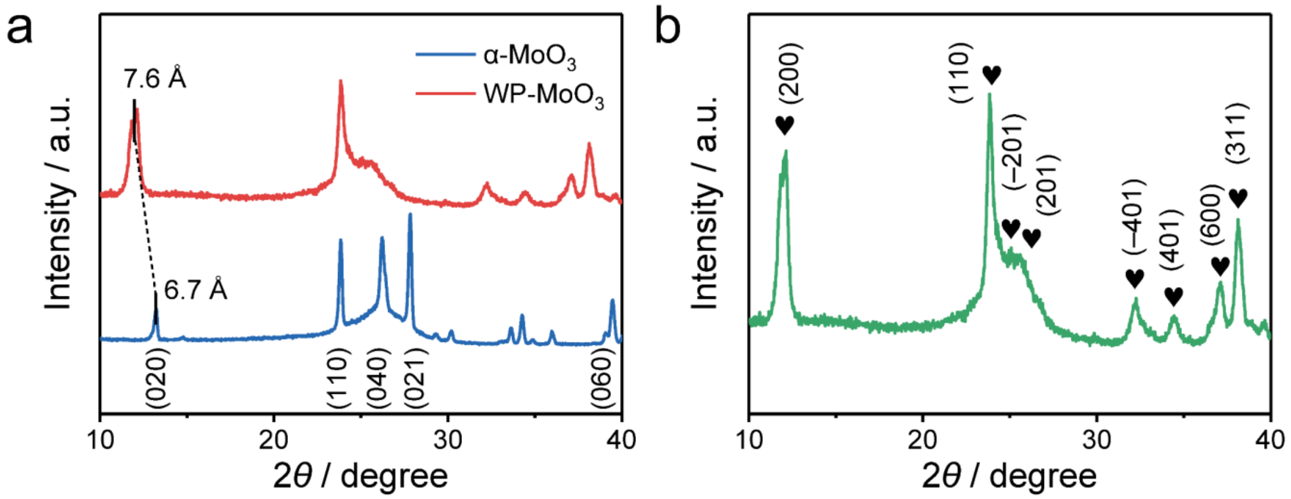

Figure S6. (a) XRD patterns of α-MoO_3 and WP-MoO_3. (b) Corresponding crystal planes of WP-MoO_3. α-MoO_3 can be fitted well with the orthorhombic α-MoO_3 (JCPDS#05-0508), with an interlayer spacing of 6.7 Å**.** The interlayer spacing of the WP-MoO_3 is 7.6 Å.

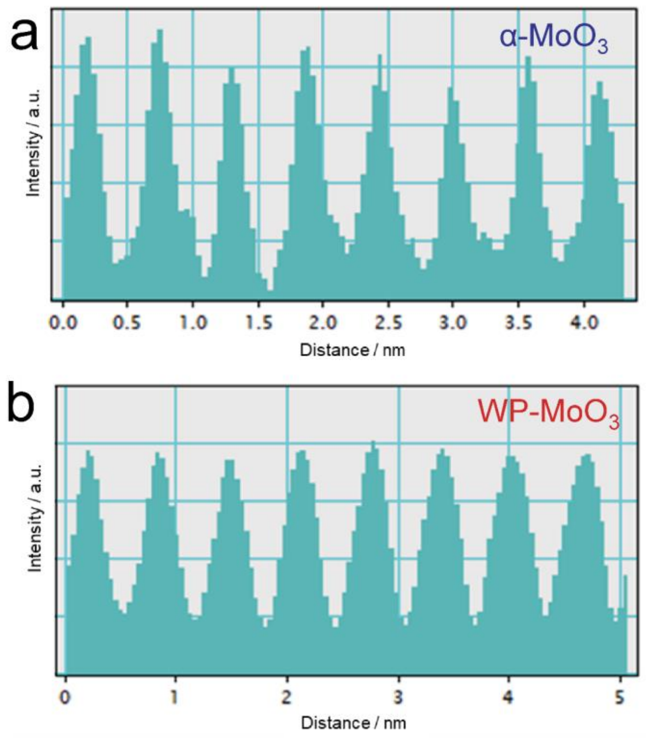

Figure S7. Contrast intensity profiles of α-MoO_3 and WP-MoO_3. The results correspond to high-resolution TEM (HRTEM) images shown in Figure 2c. The interlayer spacing of (a) α-MoO_3 and (b) WP-MoO_3 are 6.7 Å and 7.6 Å, respectively.

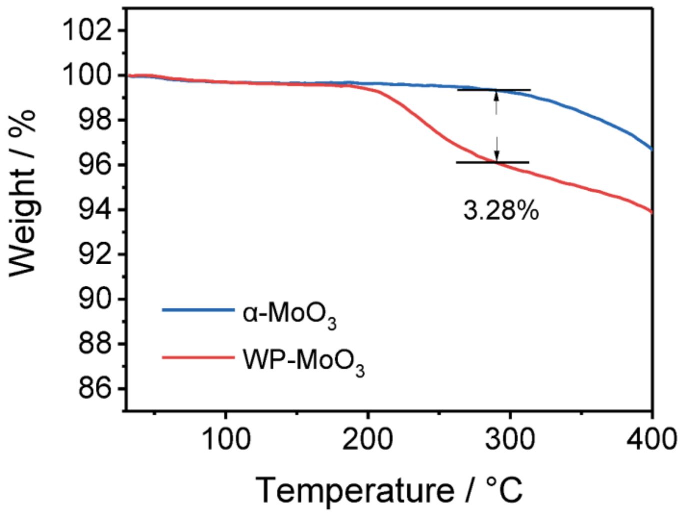

Figure S8. TGA results of α-MoO_3 and WP-MoO_3 from 30-400 °C in argon. The mass loss started at 200 °C can be attribute to the loss of the crystal water. The employed samples contain PVDF (10%) and acetylene black (10%) in TGA tests. The mass loss of PVDF and acetylene black was deducted as background. Even so, it is not a quantitative method.

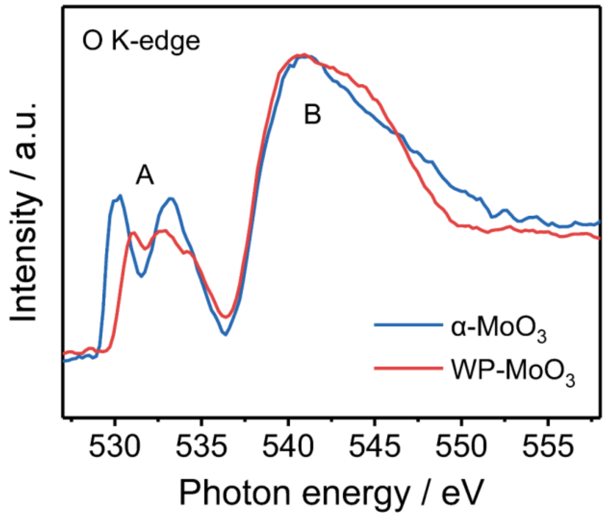

Figure S9. Normalized O K-edge XANES spectra of α-MoO_3 and WP-MoO_3. The energy region of 530-540 eV refers to the hybridization of O 2p with highly dispersive Mo 4d states. WP-MoO_3 shows the decrease of peak intensity at this energy region, which reflects less Mo unoccupied 4d states hybridizes with O 2p orbitals in WP-MoO_3 and a more electron-rich state at the Mo sites while comparing with α-MoO_3.

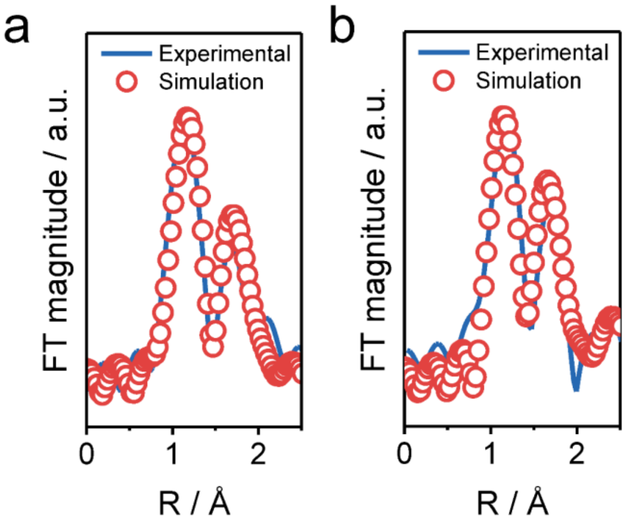

Figure S10. Radial distribution of Fourier-transformed EXAFS signal and the corresponding fitting curves of (a) α-MoO_3 and (b) WP-MoO_3.

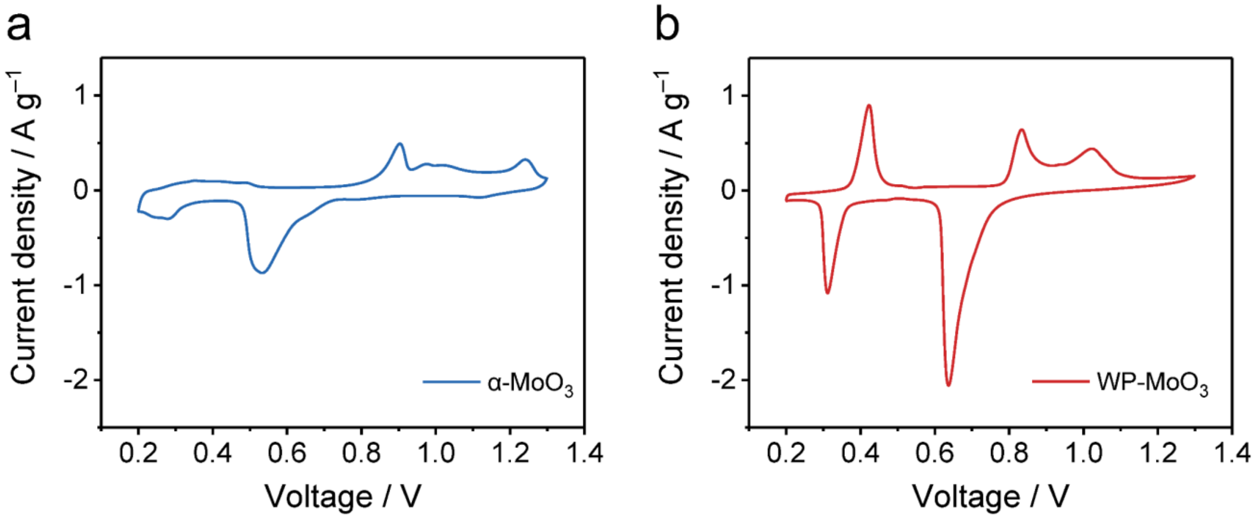

Figure S11. CV curves of α-MoO_3 and WP-MoO_3 electrodes. The CV scan results of (a) α-MoO_3 electrode and (b) WP-MoO_3 electrode at 0.7 mV s^{-1}.

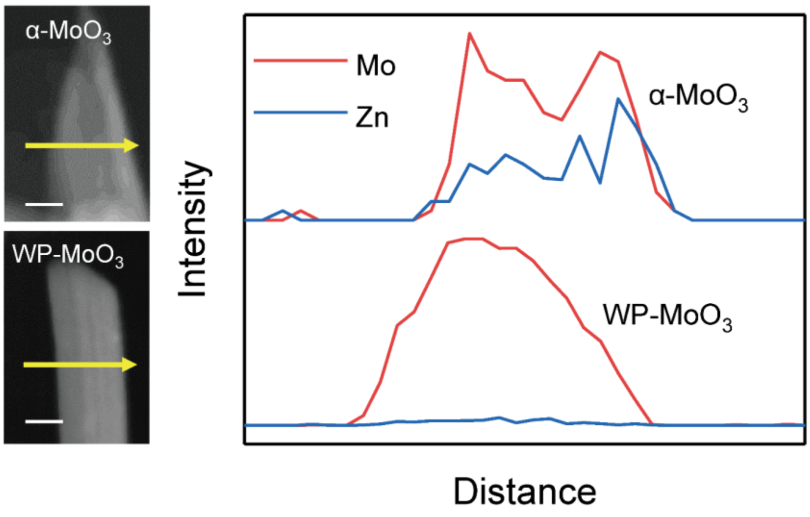

Figure S12. Linear EDX scan of Mo and Zn elements in fully discharged α-MoO_3 and WP-MoO_3. Scale bars: 50 nm.

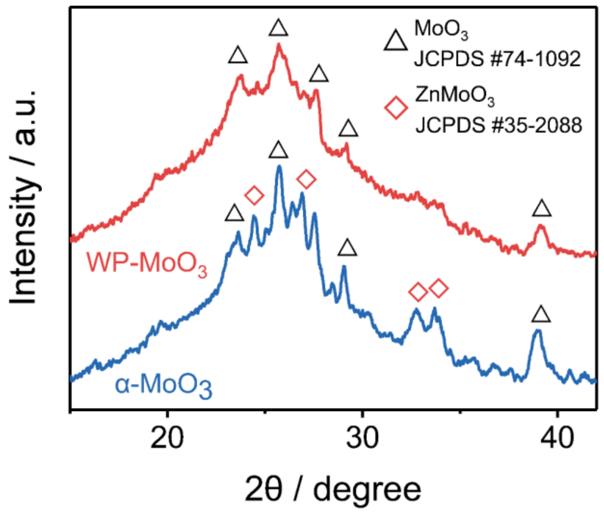

Figure S13. XRD of the fully discharged α-MoO_3 and WP-MoO_3 electrodes after annealing in air. The annealing condition is 500 °C in air for 3 h. Both MoO_3 (JCPDS#74-1092) and $ZnMoO_3$ (JCPDS#35-2088) are observed in the annealed α-MoO_3, and only MoO_3 (JCPDS#74-1092) is observed in the annealed WP-MoO_3.

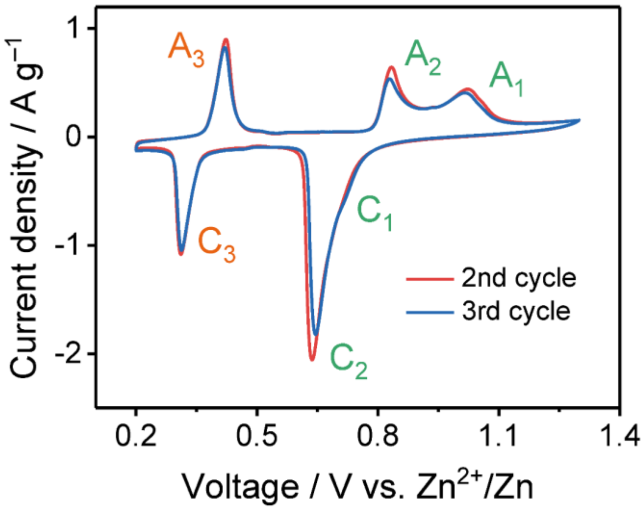

Figure S14. The second and third CV cycles of WP-MoO_3 at 0.7 mV s^{-1}. The CV curves show three anodic peaks (denoted as A_1, A_2, and A_3) and three cathodic peaks (denoted as C_1, C_2, and C_3). C_1 and C_2 overlap with each other to form a broad peak, which can be evidenced by the asymmetric peak shape.

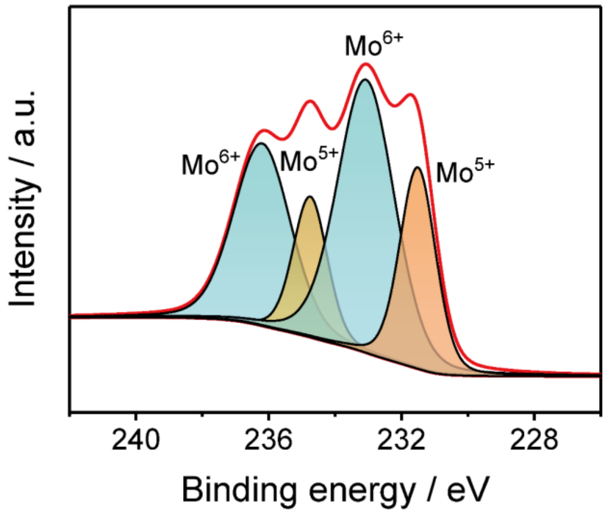

Figure S15. Mo 3d XPS spectrum of WP-MoO_3-c. Two pairs of doublet peaks can be attribute to Mo^{5+} and Mo^{6+}.

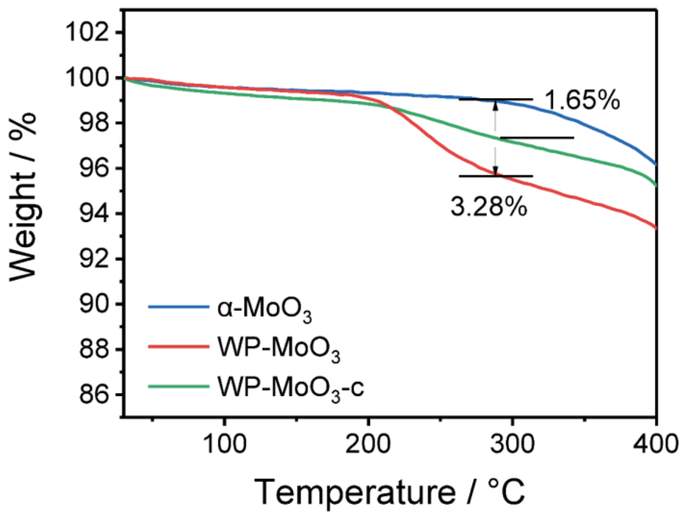

Figure S16. TGA results of WP-MoO_3-c. The result is compared with α-MoO_3 and WP-MoO_3 from 30-400 °C in argon.

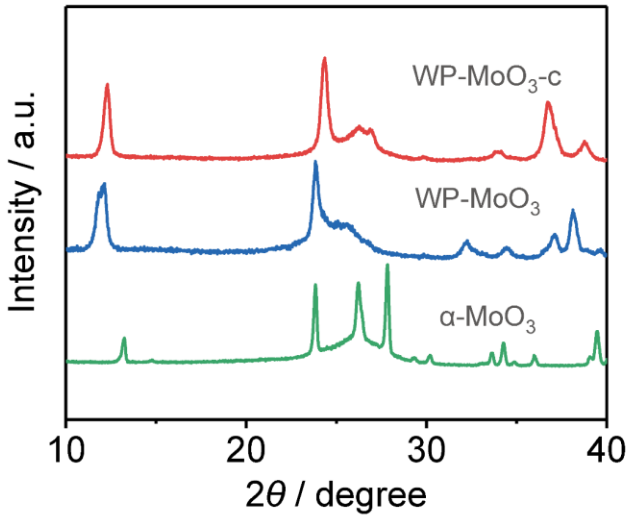

Figure S17. XRD spectra of WP-MoO_3-c. The spectra is compared with α-MoO_3 and WP-MoO_3. The calculated interlayer spacing is 7.4 Å for WP-MoO_3-c.

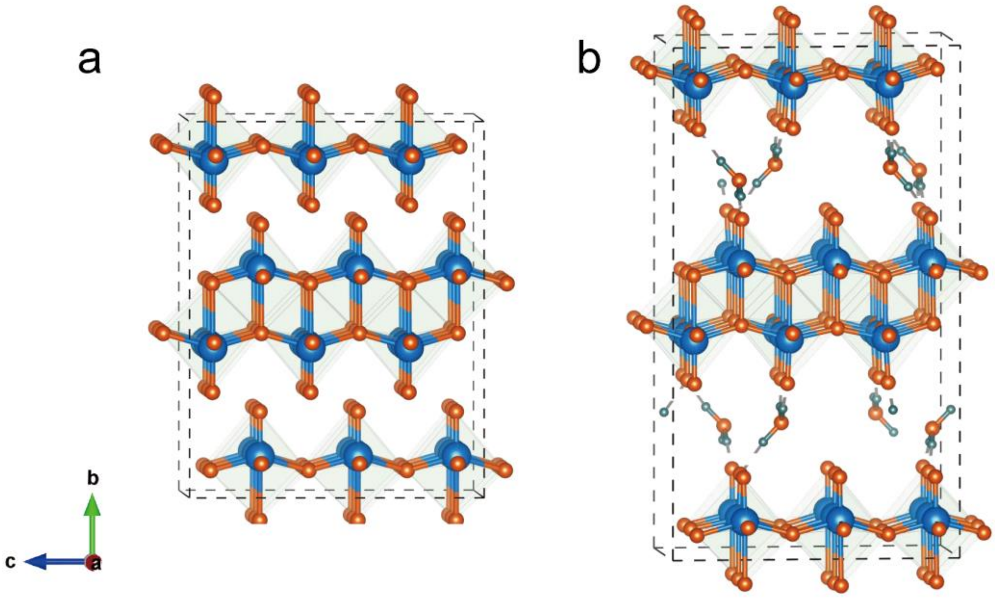

Figure S18. The simulated structures of α-MoO_3 and WP-MoO_3-c. The interlayer spacing has been obviously expanded from (a) α-MoO_3 to (b) WP-MoO_3-c.

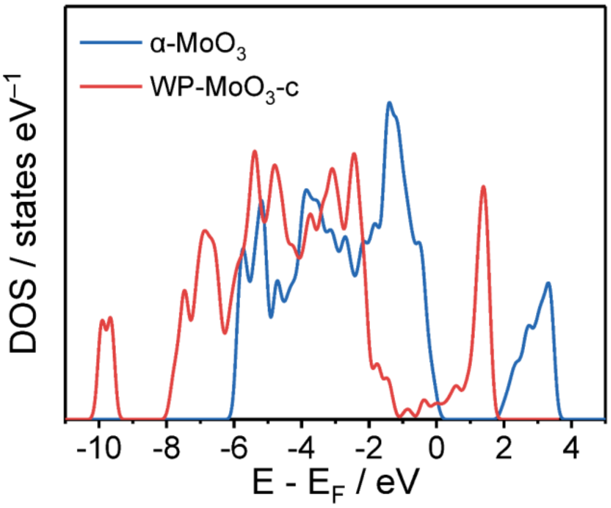

Figure S19. Calculated total density of states (TDOS) of α-MoO_3 and WP-MoO_3-c. The bandgap of α-MoO_3 and WP-MoO_3-c are 2.18 eV and 0.04 eV, respectively. The peak at −10 eV can be attribute to the formant of terminal O-p orbital with H-s orbital. The lattice expansion by water-proton insertion and the interaction between insertion species and terminal O atoms lead to the density of states shift down far away from Fermi level.

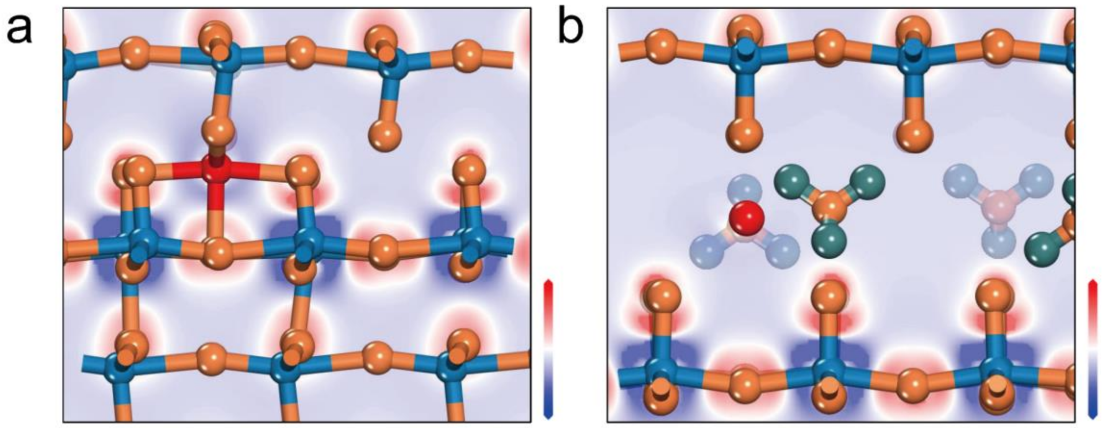

Figure S20. The side-view electron density difference of α-MoO_3 and WP-MoO_3-c after the Zn intercalation. Two samples have different coordination environments of Zn. In (a) α-MoO_3, terminal O atom in the layer cooperates with the insertion Zn. In (b) WP-MoO_3, it has nearly no interaction between Zn and terminal O atom.

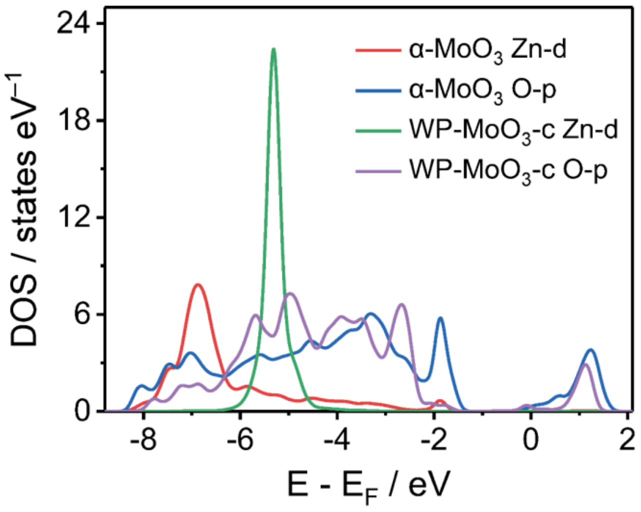

Figure S21. Projected density of states (PDOS) of Zn-d orbital and adjacent O-p orbital of α-MoO_3 and WP-MoO_3-c after the Zn intercalation. In α-MoO_3, the Zn-d orbital and the O-p orbital have obvious interaction resonance at the conductive band (especially at −2 to −8 eV), and there is no resonance in WP-MoO_3-c.

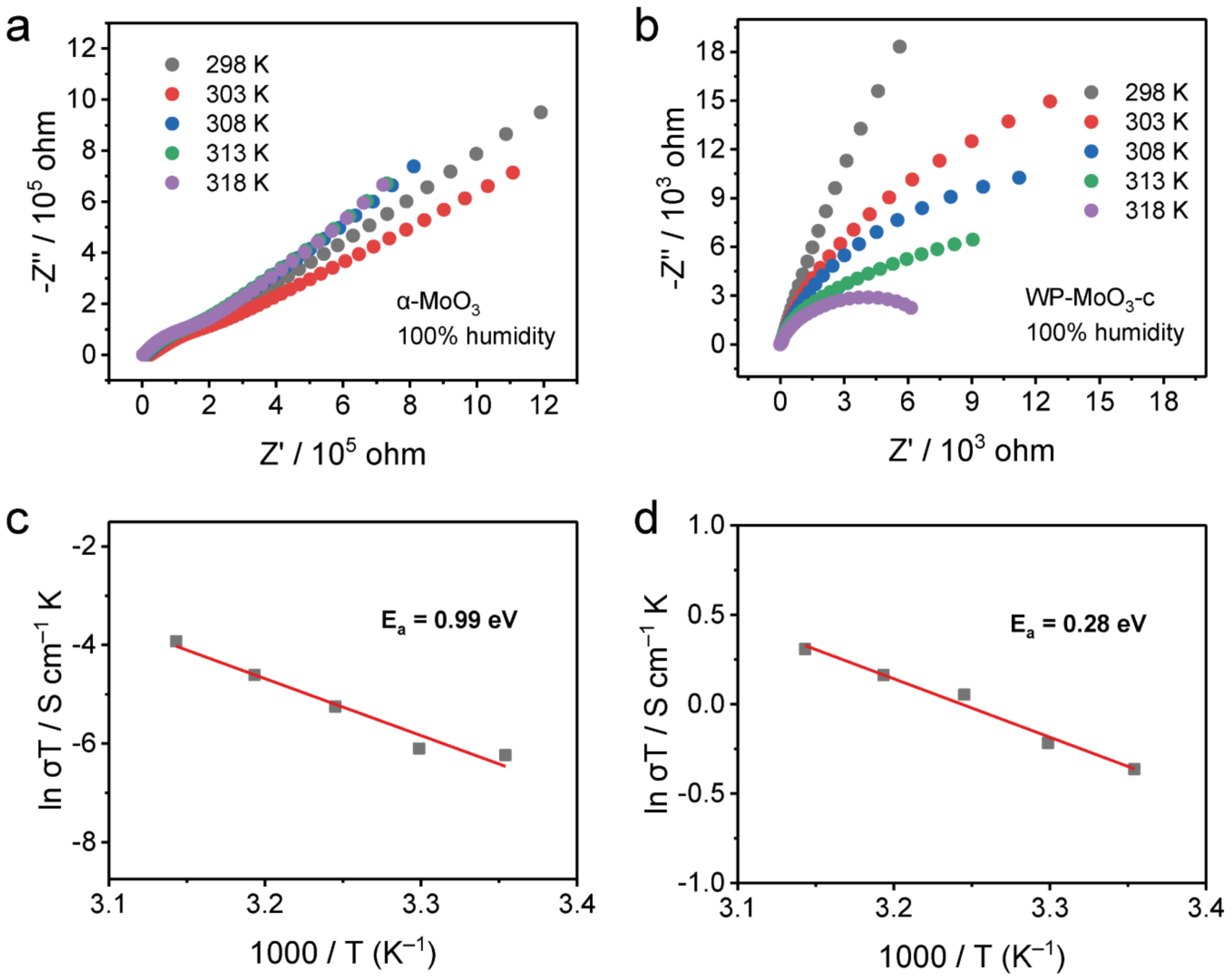

Figure S22. EIS analyses at various temperatures and 100% humidity of (a) α-MoO_3 and (b) WP-MoO_3-c samples. Arrhenius plots of (c) α-MoO_3 and (d) WP-MoO_3-c. As shown, the proton conduction activation energy (E_a) of WP-MoO_3-c was calculated as 0.28 eV, which suggests the Grotthuss conduction mechanism (E_a < 0.4 eV). In comparison, α-MoO_3 exhibits a large E_a of 0.99 eV.

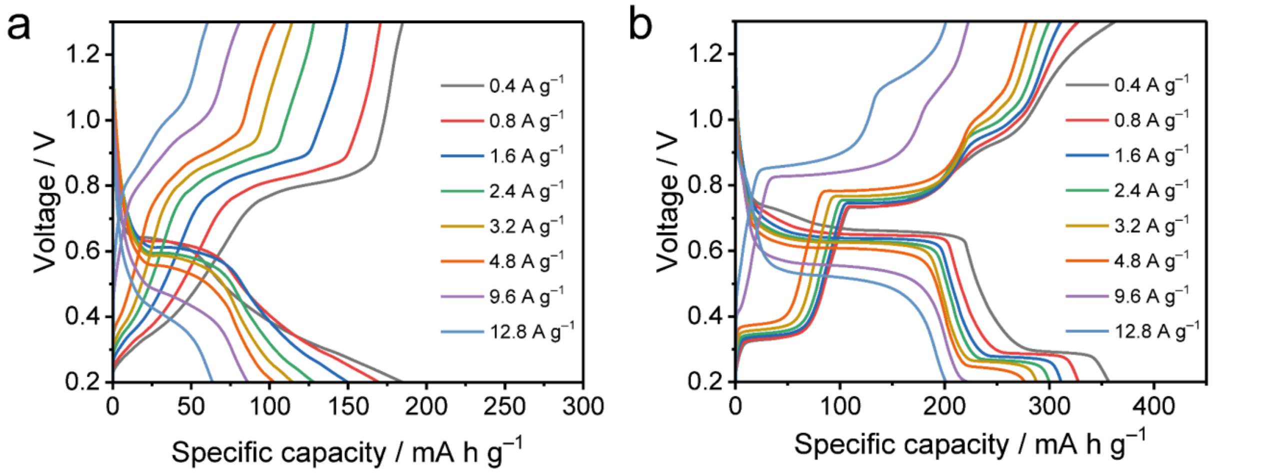

Figure S23. GCD curves of (a) Zn/α-MoO_3 and (b) Zn/WP-MoO_3 devices at various current densities. The specific capacities are based on the cathode mass.

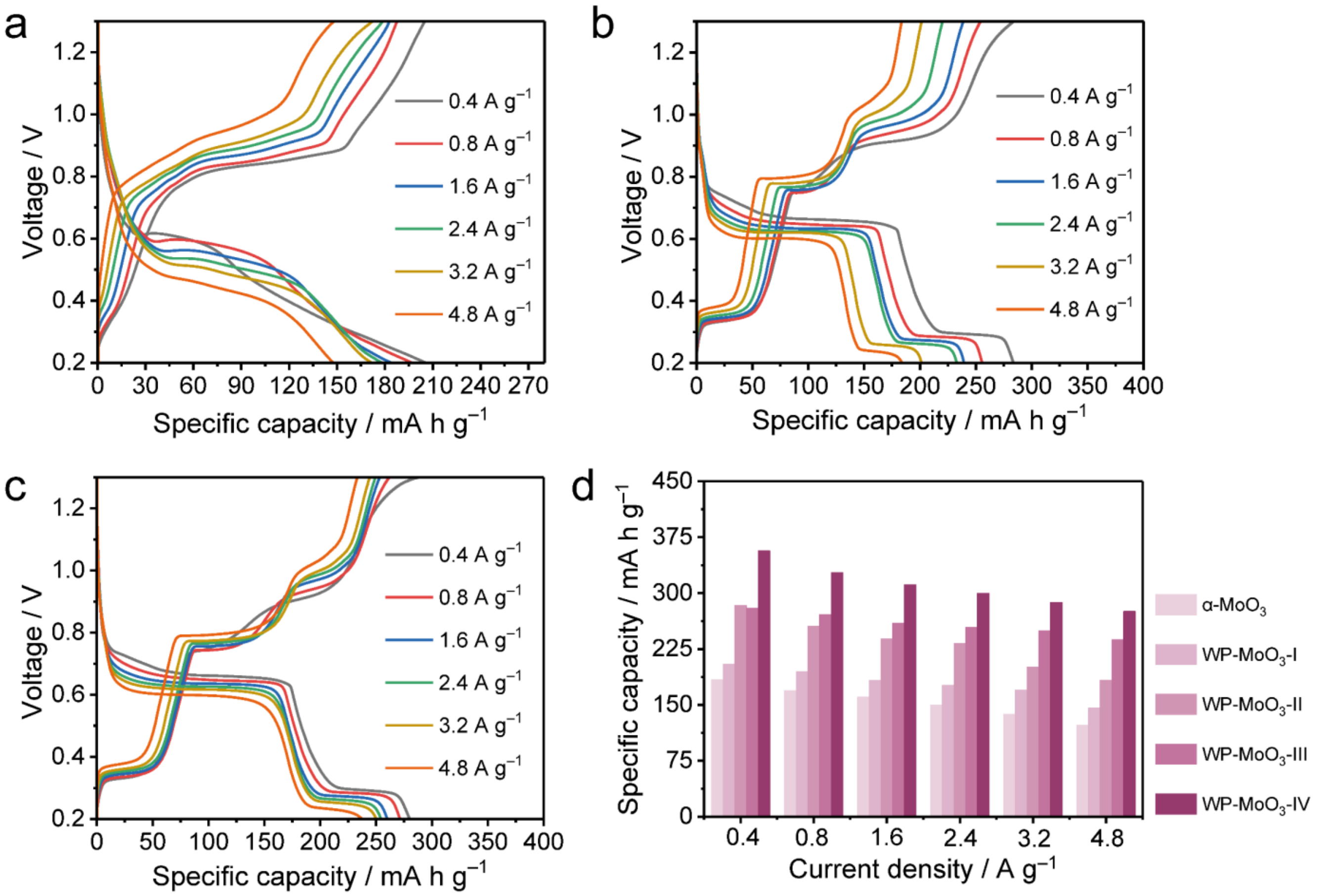

Figure S24. (a-c) GCD profiles for Zn/WP-MoO_3 devices using cathodes with different amounts of intercalated H^+. (a) Zn/WP-MoO_3-I (−0.1 V *vs.* SCE, 0.25 H^+ per MoO_3), (b) Zn/WP-MoO_3-II (−0.34 V *vs.* SCE, 1.0 H^+ per MoO_3), and (c) Zn/WP-MoO_3-III (−0.53 V *vs.* SCE, 1.25 H^+ per MoO_3). (d) Specific capacities of Zn/α-MoO_3 and Zn/WP-MoO_3 devices employing as-prepared electrodes with different cut-off potentials. The specific capacities are based on the cathode mass. The optimal WP-MoO_3 electrode was prepared with a cut-off potential of −0.8 V *vs.* SCE.

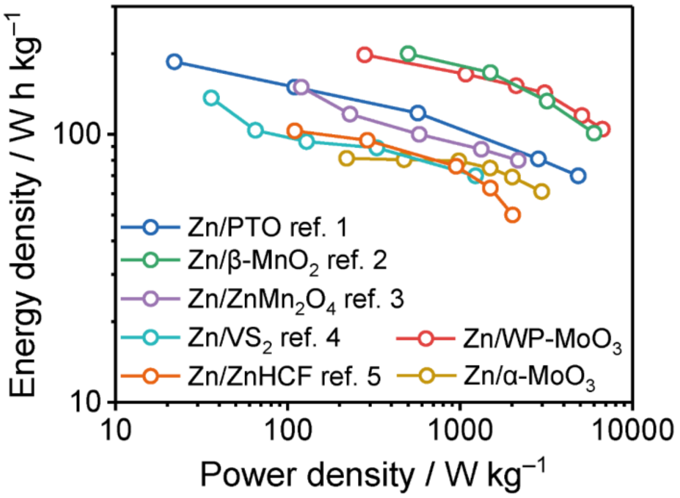

Figure S25. Ragone plots of Zn/α-MoO_3 and Zn/WP-MoO_3 devices. The performances are compared with recently reported state-of-art ZMBs.[1-5] PTO represents pyrene-4,5,9,10-tetraone.

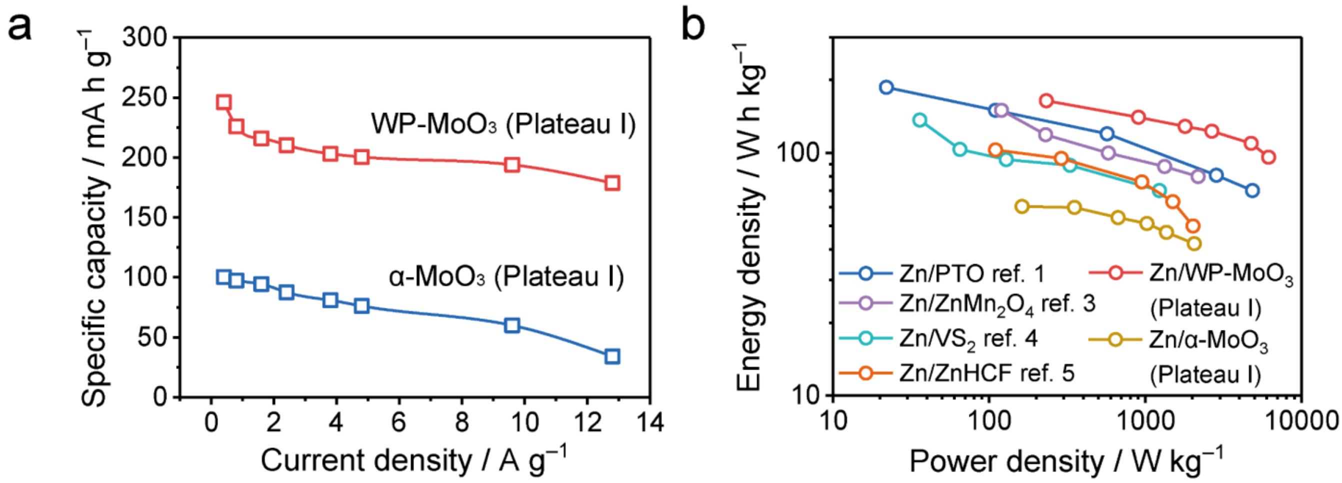

Figure S26. (a) Rate performance and (b) Ragone plots of Zn/α-MoO_3 and Zn/WP-MoO_3 at the voltage range of 0.4-1.3 V (only with plateau I). The Ragone plots are compared with recently reported state-of-the art ZMBs.[1,3-5] PTO represents pyrene-4,5,9,10-tetraone.

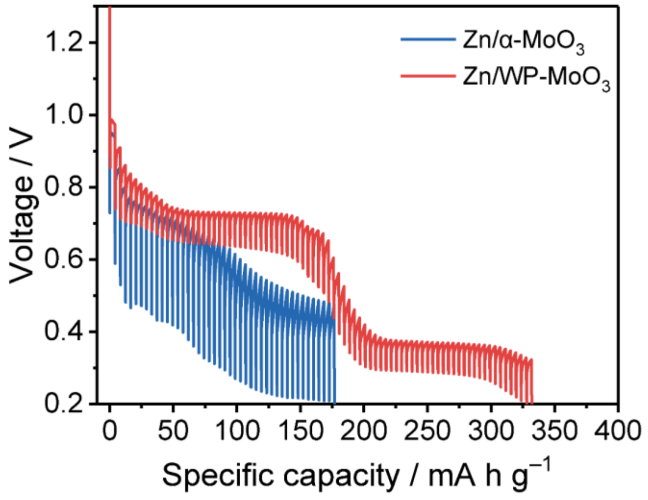

Figure S27. GITT curves of Zn/α-MoO_3 and Zn/WP-MoO_3.

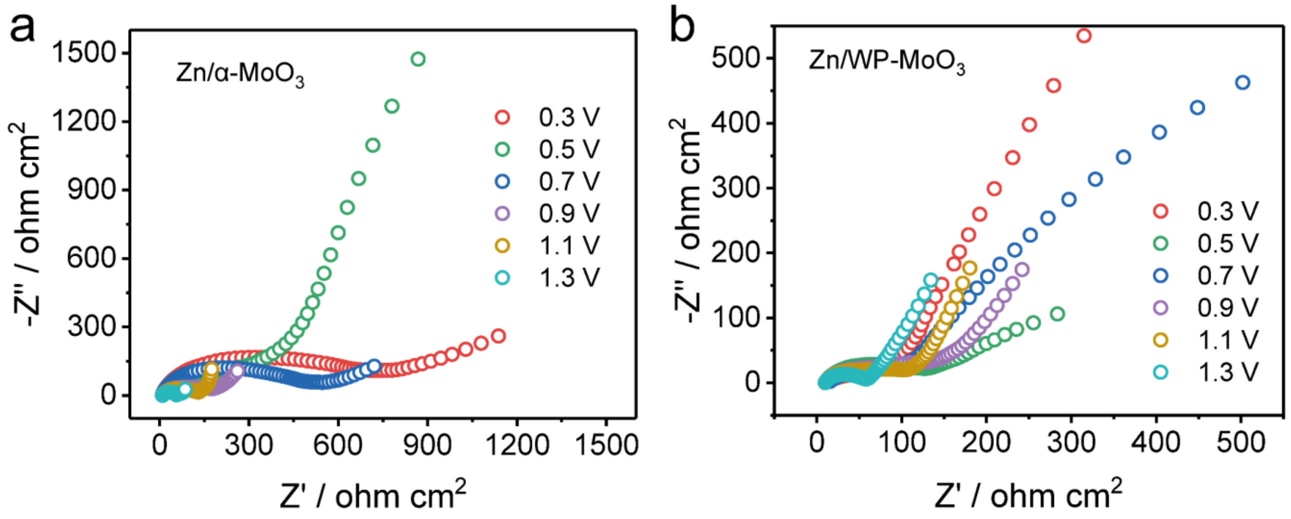

Figure S28. Nyquist plots of Zn/α-MoO_3 and Zn/WP-MoO_3 at different voltages. (a) Zn/α-MoO_3; (b) Zn/WP-MoO_3.

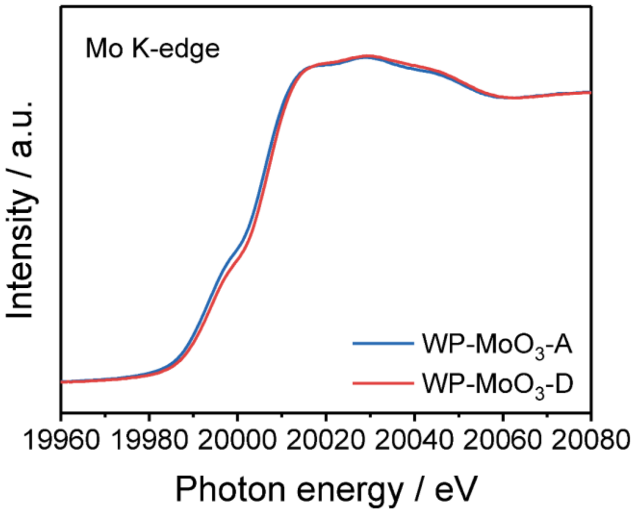

Figure S29. Normalized Mo K-edge XANES spectra of WP-MoO_3 at the fully charged and discharged state. Fully charged state corresponding to the 1.3 V (WP-MoO_3-A), and fully discharged state corresponding to the 0.2 V (WP-MoO_3-D).

Table S1. EXAFS fitting parameters of the Mo K-edge for α-MoO_3 and WP-MoO_3. (S_0^2=0.932)

Sample	Shell	N^a	R(Å)^b	σ^2(Å^2)^c	ΔE_0(eV)^d	R factor
α-MoO_3	Mo=O	2.0	1.72	0.0031	5.9	0.021
	Mo−O	1.8	1.96	0.0031		
WP-MoO_3	Mo=O	1.4	1.73	0.0031	4.6	0.029
	Mo−O	1.4	1.97	0.0031		

$^a N$: coordination numbers; $^b R$: bond distance; $^c \sigma^2$: Debye-Waller factors; ^{d} ΔE_0: the inner potential correction. R factor: goodness of fit, according to the experimental EXAFS fit of Mo foil reference by fixing the coordination number of Mo-Mo to be 8 and 6 as the known crystallographic value.

Table S2. Summary of resistance (*R*), proton conductivity (*σ*) and activation energy (E_a) of α-MoO_3 and WP-MoO_3-c.

		298 K	303 K	308 K	313 K	318 K	E_a / eV
α-MoO_3	*R* / Ω	23400	20786	9037	4831	2486	0.99
	σ / S cm^{-1}	6.6×10^{-6}	7.4×10^{-6}	1.7×10^{-5}	3.2×10^{-5}	6.2×10^{-5}	
WP-MoO_3-c	*R* / Ω	66	58	45	41	36	0.28
	σ / S cm^{-1}	2.3×10^{-3}	2.7×10^{-3}	3.4×10^{-3}	3.8×10^{-3}	4.3×10^{-3}	

Author Contributions

X. Lu, M. Y. and H. Z. planned and designed the project. H. Z. and W. W. fabricated the materials and performed the electrochemical experiments. Q. L. performed and analyzed the EIS result. X. S. carried out the TEM measurements. F. Y. conducted the DFT analysis. X. Liu contributed to the important discussion. X. Lu, M. Y. and H. Z. analyzed the data and wrote the manuscript. All authors discussed the results and commented on the manuscript.

Reference

[1] Z. Guo, Y. Ma, X. Dong, J. Huang, Y. Wang, Y. Xia, *Angew. Chem. Int. Ed.* **2018**, *57*, 11737-11741.
[2] N. Zhang, F. Cheng, J. Liu, L. Wang, X. Long, X. Liu, F. Li, J. Chen, *Nat. Commun.* **2017**, *8*, 405.
[3] N. Zhang, F. Cheng, Y. Liu, Q. Zhao, K. Lei, C. Chen, X. Liu, J. Chen, *J. Am. Chem. Soc.* **2016**, *138*, 12894-12901.
[4] P. He, M. Yan, G. Zhang, R. Sun, L. Chen, Q. An, L. Mai, *Adv. Energy Mater.* **2017**, *7*, 1601920.
[5] L. Zhang, L. Chen, X. Zhou, Z. Liu, *Adv. Energy Mater.* **2015**, *5*, 1400930.
